# Fusobacterium periodonticum BCT protein targeting glucose metabolism to promote the epithelial-mesenchymal transition of esophageal cancer cells by lactic acid

**DOI:** 10.1186/s12967-024-05157-z

**Published:** 2024-04-30

**Authors:** Xinxin Guo, Ping Wan, Weitao Shen, Mingjun Sun, Zhenyan Peng, Yinghao Liao, Yang Huang, Ran Liu

**Affiliations:** Key Laboratory of Environmental Medicine Engineering, School of Public Health, Ministry of Education, Southeast University, 87 Dingjiaqiao Street, Nanjing, 210009 China

**Keywords:** Fusobacterium periodonticum, Esophageal cancer, Glycolysi, TLR4/Akt/HIF-1α, EMT

## Abstract

**Background:**

The cancer microbiota was considered the main risk factor for cancer progression. We had proved that Fusobacterium periodonticum (F.p) was higher abundance in Esophageal cancer(EC)tissues. Bioinformation analysis found that BCT was a key virulence protein of F.p. However, little is known about the role and mechanism of BCT in EC. This study aimed to recognize the key virulence protein of F.p and explore the mechanism of BCT in promoting EC.

**Methods:**

We constructed a eukaryotic expression vector and purified the recombinant protein BCT. CCK8 used to analyzed the activity of EC after treated by different concentration of BCT. UPLC-MS/MS and ELISA used to detect the metabonomics and metabolites. The ability of migration and invasion was completed by transwell assay. RT-QPCR, WB used to analyze the expression of relevant genes.

**Results:**

Our data showed that BCT was higher expression in EC tumor tissues (*p* < 0.05) and BCT in 20 µg/mL promoted the survival, invasion and migration of EC cells (EC109) (*p* < 0.05). Meanwhile, UPLC-MS/MS results suggested that BCT resulted in an augmentation of hypotaurine metabolism, arachidonic acid metabolism, glycolysis/gluconeogenesis, tryptophan metabolism, citrate cycle activity in EC109. The metabolic changes resulted in decreasing in glucose and pyruvate levels but increase in lactate dehydrogenase (LDH) activity and lactic acid (LA) as well as the expression of glucose transporter 1, Hexokinase 2, LDH which regulated the glycolysis were all changed (*p* < 0.05). The BCT treatment upregulated the expression of TLR4, Akt, HIF-1α (*p* < 0.05) which regulated the production of LA. Furthermore, LA stimulation promoted the expression of GPR81, Wnt, and β-catenin (*p* < 0.05), thereby inducing EMT and metastasis in EC109 cells.

**Conclusion:**

Altogether, these findings identified that impact of BCT in regulation of glycolysis in EC109 and its involves the TLR4/Akt/HIF-1α pathway. Meanwhile, glycolysis increasing the release of LA and promote the EMT of EC109 by GPR81/Wnt/β-catenin signaling pathway. In summary, our findings underscore the potential of targeting BCT as an innovative strategy to mitigate the development of EC.

**Supplementary Information:**

The online version contains supplementary material available at 10.1186/s12967-024-05157-z.

## Introduction

Cancer is still the second-leading cause of mortality in the Global [1–3]. In the face of global trends, the burden of cancer on the aging population is increasingly alarming. Notably, there has been a significant rise in the incidence of various cancers among young adults over recent decades. Over the past few decades, the incidence of various cancers among young adults has been a notable increase [4]. The development of cancer involves a complex process encompassing genetic inheritance, environmental factors, unhealthy lifestyles, and microorganisms [5–7].. Therefore, effective interventions and treatments can play a crucial role in reducing both cancer occurrence and mortality rates while alleviating the associated burden.

Esophageal cancer (EC) is a significant global health challenge, characterized by elevated incidence and mortality rates attributed to its multifactorial etiology and advanced stage at diagnosis [8]. Meanwhile, currently limited level of treatment including chemotherapy, immunotherapy, hormone therapy surgery, radiotherapy and polymeric nano-medicines could not control the development of cancers effectively [9, 10]. Numerous studies have acknowledged that patients experience various adverse effects depending on their treatment experiences [11, 12]. For instance, as the most common choice for caner therapy, chemo-therapy not only kill cancer cells and the normal tissues. Meanwhile, chemotherapy also alters patients’ microbiome involvement in tumor regulating [12]. Consequently, combination therapy has been proposed as a means to enhance the therapeutic efficacy in cancer management.

Huai’an city in Jiangsu province is among the regions with high incidence rates of EC in China. The occurrence of EC is a multifaceted process influenced by genetic inheritance, environmental factors, and microorganisms, involving various factors, stages, and steps [5]. Our investigation into the etiology of the elevated incidence of EC in Huai’an has revealed that suboptimal oral hygiene significantly contributes to this phenomenon. Furthermore, previous studies have consistently demonstrated an association between inadequate oral hygiene and an increased risk of EC. Our investigation into the etiology of the elevated incidence of EC in Huai’an has revealed that suboptimal oral hygiene significantly contributes to this phenomenon. Furthermore, previous studies have consistently demonstrated an association between inadequate oral hygiene and an increased risk of EC [13, 14]. Our previous study also revealed a significant disparity in the microbial composition between tumor and para-tumor tissues, particularly with regards to the higher abundance of Fusobacterium periodonticum (F.p) in tumor tissue compared to adjacent tumor tissue as determined by 16 S rDNA sequencing [15]. Microorganisms are involved in the development of many diseases, such as Fusarium induced inflammatory disorders [16], and the Fusobacterium nucleatum regulated the occurrence and development of cancers [17, 18]. Although the F.p was been founded high infiltrated in the EC tissues, the role of F.P in EC was still unknown.

F.p was a genus of absolutely anaerobic filamentous gram-negative rods, and it was a typical oral pathogenic bacteria [19], which obtained from periodontitis lesions in patient with insulin-dependent diabetes mellitus. Recent advancements in next-generation sequencing technology have revealed that F.p can infiltrate tumor tissues, such as colon cancer, oral cancer, and pancreatic cancer. Furthermore, it has been implicated in the development and progression of various diseases across different tissue types [20–24]. In addition, Sulit A K, et al. suggested that the Lps deriver from F.p in the colorectal cancer promote the produce of CCL20 to regulate tummor immunity and influenced the prognosis of colorectal cancer [25]. However, direct and conclusive evidence regarding the impact of F.p on EC progression remains elusive, and the underlying mechanisms by which F.p contributes to cancer development have yet to be fully elucidated. Notable, a key virulence characteristic of F.p is its capacity for adhesion and colonization in various mammalian cells. A study has suggested that fibroblast activation protein 2 (Fap2) as a galactose-sensitive hemagglutinin adhesion, playing a crucial role as an out-surface virulence protein of fusobacterial such as Fusobacterium nucleatum (F.n), which has been demonstrated to promote cancer progression [26]. The F.p contains a sequence resembling FAP2, which has been designated as BCT. However, the role and mechanism of the virulence potential BCT in the evolution of EC have not yet been investigated.

Recently, accumulating evidence has indicated that metabolic reprogramming of tumors represents a fundamental characteristic of tumorigenesis [27]. In particula glycolysis, serving as the primary mode of energy supply, plays a pivotal role in meeting the elevated metabolic energy demands of cancer cells, and thus a significant indicator of malignant tumor transformation [28, 29]. However, the metabolic reprogramming was regulated by majority factors. For instance, the insulin promotes the conversion of excess glucose into glycogen [30]. In ESCC, metabolic reprogramming has also been observed with distinct alterations in glycolysis, anaerobic respiration, and protein and lipid metabolism within the circulating metabolisma [31, 32]. The metabolic reprogramming of patients with esophageal cancer is also susceptible to the influence of exogenous substances such as curcumin, trashinone, tabacoco, et al [33–35]. As is well known, TLR4 plays a crucial role an important receptor for pathogen [36]. Recent studies have provided evidence of the pivotal involvement of TLR4 in glucolipid metabolism, as evidenced by its ability to enhance HIF-1α expression and promote glycolysis [37]. HIF-1α is deemed a crucial transcription factor owing to its pivotal roles in regulating glycolysis, angiogenesis, cellular differentiation, apoptosis and other vital pathways [38]. Tumor cells are known to exhibit a high rate of glucose consumption through glycolysis, resulting in the production of pyruvate that is subsequently metabolized into lactic acid (LA) by lactate dehydrogenase (LDH), ultimately leading to its release into the tumor microenvironment (TME). The acidic environment caused by LA accumulation promotes tumor progression and metastasis by EMT [39], since LA could activate the GF-β/Smad、Wnt/β-catenin、IL-6/STAT3 and HGF/MET pathway inducing EMT [40, 41]. Despite accumulating evidence demonstrating higher F.p levels in EC tissues, whether its virulence protein BCT mediates EC progression through metabolic reprogramming and related mechanisms remains unknown. However, further investigation is required to determine if metabolic reprogramming in EC is associated with F.p infiltration and elucidate its regulatory mechanism.

In the current study, we identified the virulence protein of F.p-BCT and successfully constructed the recombinant protein. Metabolic changes in EC109 cells treated by BCT were analyzed using UPLC-MS/MS, enabling us to detect key products of glycolysis and elucidate the underlying mechanism by which BCT promotes glycolysis in EC109. Additionally, Our findings revealed that activation of TLR4/Akt/HIF-1α is involved in BCT-induced release of lactate (LA) from EC109 cells, and LA further promotes epithelial-mesenchymal transition (EMT) via GPR81/Wnt/β-catenin signaling pathway. Importantly, our data provide novel insights into the metabolic reprogramming caused by F.P virulence protein BCT that may contribute to EC pathogenesis.

## Materials and methods

### Samples and cells

We enrolled a total of 38 EC patients from First People’s Hospital of Huai’an, Jiangsu Province, China, from July 2020 to December 2020. The inclusive criteria for the patients were initial diagnosed and prior therapy. Detail information is provided in Table 1. This research proposal was reviewed and approved by the ethics committee of Zhongda Hospital of Southeast University, and the grant number is 2021ZDKYSB004. The Esophageal cancer cell (EC109) was purchased from Chinese Academy of Sciences Cell Bank (Shanghai, China) and cultured in humidified atmosphere at 37 °C with 5% CO_2_ using 1640 medium (Gibco, USA), supplemented with 10% Fetal Bovine Serum (ExCell, USA), as well as 100 U/mL penicillin and 100 U/mL streptomycin (Solarbio, China).

**Table 1:** Detail information of EC patients and health people

Features	EC(*n* = 38)	Health(*n* = 38)
Age(x̅ ± S)	66.3 ± 4.7	64.9 ± 4.0
gender		
male	30(78.9%)	30(78.9%)
femal	8(21.1%)	8(21.1%)
Somking		
Yes	23(60.5%)	20 (52.6%)
No	15(39.5%)	18(47.4%)
Drinking		
Yes	18 (47.4%)	18 (47.4%)
No	20(52.6%)	20(52.6%)
Stage		
I	2 (5.3%)	-
II	4 (10.5%)	-
III	23 (60.5%)	-
None	9 (23.7%)	-
differentiation		
Low differentiation	4 (10.5%)	-
Middle differentiation	24 (63.2%)	-
High differentiation	2 (5.3%)	-
None	8 (21.0%)	-
Metastasis		
Yes	19 (50.0%)	-
No	15 (39.5%)	-
None	4 (10.5%)	-

### DNA extraction

DNA extraction Kit (TIANGEN, China) was used to extract DNA. A total of 20 µg EC tumor and para-tumor tissue were added to GA buffer solution and grinded with tissue grinding machine (jinxin, China). Subsequently, 20µL proteinase K used to lysed the tissues at 56℃ for 3 h. The extraction steps followed the provided protocol. After complete dissolution in 200µL GB buffer solution at a temperature of 70℃ in water bath for 10 min until the solution became clear, an additional volume of 200µL absolute ethyl alcohol was added. Precipitation was collected by adsorption column following centrifugation at a speed of 13,000 g for 30s. This was followed by washing with 500µL GD and 600µL PW solution. Finally, TE buffer was used to dissolve the DNA and its concentration was determined using the ND-1000 microultraviolet spectrophotometerto (Thermo, USA).

### Quantitative analysis of BCT by RT-QPCR

RT-QPCR was employed to determine the relative expression of BCT in EC tissues and para-tissues. The reaction system followed the protocol of Vizyme, China. The PCR procedure was 95℃ for 3 min, 95℃ for 15s, 60℃ for 30s, 72℃ for 30s, (40 cycles), 72℃ for 10 min. 2^−ΔCT^ was used to react the BCT relative abundance and 16 S as the reference gene. Primer sequences are listed in Table 2.

**Table 2:** Primer sequence

Genes name	Forward-5’	Reverse-5’
BCT	GCTCCAACAGCTCCAACAGTA	GGTAGGACTGCTTTACCCGTAG
16 S	ACTCCTACGGGAGGCAGCAG	GGACTACHVGGGTWTCTAAT

### Construct BCT recombination protein by prokaryotic expression system

We obtained the sequence of BCT from NCBI and the gene was synthesized by Sangon. pET − 28α expression vector and BCT was restriction enzyme digested by NdeI and XhoI. pET-28α-BCT plasmids were recovered after electrophoresis. After mixing according to 4:1 substance ratio, add 5 µL solution I at 16℃ for a night. The next day, the plasmid was transfered into escherichia coli BL5. After screening by kana plate, the plasmid was extracted from single colony and the sequence was done by Sangon Biotech. The pET-28α-BCT plasmid was transfered into escherichia coli BL5. The constructed single colony of escherichia coli BL5 containing pET-28α-BCT plasmid was activated by shaking overnight, and then added to 200mL LB medium with 200 µL kana at a ratio of 1:100 next day. After the OD600 of bacterial solution reached 0.6–0.8 (logarithmic growth phase) at 37℃ 220 r/min, 1 mL pre-induction bacterial was taken as control, and inducer IPTG was added into the final concentration of 0.8 mmol/L, and the induction condition was 37℃ 220 r /min. Bacteria were taken at 1, 2, 3, 4, 5 and 6 h after induction. After ultrasonic crushing, supernatant and precipitate were retained. The supernatant was purified by purification kit, and the protein products obtained after purification were quantified by BCA method. SDS-PAGE gel scan was used to verify the expression of pET-28α-BCT protein.

### Metabolomics analysis

We analyzed the metabolomics profile of intracellular and cellular supernatants. Logarithmic stages EC109 growing in 15 mm cell culture dish. 10 µg/mL, 20 µg/mL, 40 µg/mL BCT used to treated the cells for 24 h. Added 2mL methanol to treat cells after washed by PBS 3times and then scrape it off with a cell scraper. Meanwhile, cell free supernatant was collected and treated with methanol at 1:4. Centrifuged at 13,000 rpm for 20 min at 4℃ after ultrasound 15 min and stood for 10 min. Cells and cellular supernatants were all dissolved the sediment with acetonitrile and methanol (7:3) and filtered with a 0.22 μm needle filter. At the same time, we took 20µL of each sample and mixed it as the quality control sample. The metabolomics profile analysis was done using a SCIEX Triple TOF 5600 system (SCIEX, USA) with a Waters ACQUITY UPLC BEH C18 column (1.7 µM particles, 2.150 mm column, Waters, Milford, MA) at the column oven temperature 40℃. The mobile phase A was ACN/H2O (6:4, v/v, 10 mM ammonium formate, 0.1% formic acid) and B was IPA/ACN (9:1, v/v, 10 mM ammonium formate, 0.1% formic acid). The flow rate was 0.3 mL/min and the time of duration was 21 min at a 5µL injection volume. Positive and negative ion scanning mode was used for sampling quality spectrum signal acquisition. The gradient elution procedure is shown in Table 3. Ion source temperature was 200 °C and transmission line temperature was 200 °C, and the scanning mass to charge ratio range was (m/z) 50 ∼ 1000AMU.

We collected the raw data with UNIFI 1.8.1 software, and the data were imported into progenesis QI v2.3. All the models were treated with UV scaling. Compounds were identified based on exact mass numbers, secondary fragments, and isotopic distributions, while qualitative identification utilized HMDB and lipidmaps (V2.3) and METLIN databases. Partial least squares discriminant analysis (SPLS-DA) was performed with Metaboanalyst software. Univariate statistical analyses encompassed t-test and multiple of variation analysis, awith volcano maps generated using R software. Differential metabolites were analyzed through PLS-DA, T-test, and fold difference calculations followed by correlation analysis to enrich metabolic pathways.

**Table 3:** Gradient elution procedure

Time(min)	phase A (%)	phase B(%)
0	90	10
10	10	90
12	10	90
17	40	20
19	0	10
21	0	10

### CCK8 detect the cell viability

Cell viability was measured using CCK8 kit (Cellcook, China). EC109 cells was growing in 96 wells (1 × 10^3^cells/well) were treated with BCT (20 µg/mL) and inhibitors (TAK, LY294001, 2-ME)(MCE, China) or LA(Sigma, USA) for 24 h. The supernatant was instead by CCK8 (0.5 mg/mL), and then the 96 plate was incubated at 37 °C for 2 h and measured at 490 nm.

### Transwell assay the invasion and migration for EC109

The melted matrigel gel (Corning, USA) was mixed with 1640 serum-free medium at a ratio of 1:8, and 50uL of the mixture was evenly spread into the upper chamber of the transwell (Corning, USA) utill the gel solidified. A total of 3 × 10^5^ EC109 cells pretreated with BCT and inhibitor or LA werecultured in the upper chamber for migration assay and chemotaxis without matrigel. In the lower chamber, 600µL complete medium containing 20% FBS was added and incubated at 37℃ in 5% CO_2_ for 24 h. After removing non-migrating cells from the upper chamber by wiping and washing with PBS, then fixed with methanol for 10 min and stained with crystal violet for 15 min. The stained cells were observed under a microscope (Zeiss, Germany) after washed the dye with PBS. Five randomly selected fields of view were were analyzed to determine cell numbers per field.

### Enzyme-linked immunosorbent assay (ELISA) analyze the LA

The production of LA was analyzed according the ELISA kit (MeiMian, China). 50µL cell-free supernatant collected from different pretreated cells were addedto pre-coated 96-well plates with specific antibodies and incubated for 1 h at 37℃. The wells were then washed with scrubbing solution 5 times for 5 min. Conjugate reagent (50µL) was added to each well, and the plate was incubated at 37℃for another 30 min. Repeat washing 5 times for 5 min each time with scrubbing solution. After repeating the washing step five times, absorbance at a wavelength of 490 nm was measured within ten minutes after adding A and B (50µL) and 50µL stop solution. The concentration of LA was calculated based on standard curves.

### Enzyme standard method assay the pyruvix acid, glucose, LDH

We collected cell free supernnatants of EC109 which treated by BCT and different inhibitor. 5µL supernnatants or 5µL different concentrations standards of glucose and 180µL Glucose Assay Regent were added into 200µL centrifuge tube and react at 95℃ for 10 min. Absorbance was measured at 650 nm by drawing 180 µl of the mixture. The value of glucose was calculated by standard cures. 75µL supernnatants or 75µL different concentrations standards of pyruvic acid and 25 reagent 1 (Jiancheng, China) were added into 96 wells and reacted 2 min, then added 125µL reagent 2 into every well and the absorbance was measured at 520 nm. The concentration of pyruvic acid was calculated by standard cures.

LDH was measured using Lactate dehydrogenase assay kit (Jian Cheng, China). 3 × 10^3^ cells growing in 96 wells for 24 h in 37℃, 5%CO_2_. Different concentration BCT and inhibitor was treated the cells for 24 h. After 150uL LDH releasing reagent diluted 10 times with PBS was added, the reagent was incubated at 37℃ for 1 h, and then 120uL of cell supernatant from each well was carefully absorbed and added to the corresponding well in a new 96-well plate. The INT solution (10×) was diluted with INT diluent to INT (1×) solution according to the protocol. Under the condition of avoiding light, lactic acid solution: INT (1×) solution: enzyme solution = 1:1:1 LDH detection working solution. 60uL of LDH test working solution was absorbed and added to each well to be tested. The 96-well plate incubated on the horizontal shaker for 30 min in the dark, and the absorbance was measured at 490 nm.

### Short interfering RNA (SiRNA) transfection

Before plasmids transfection, EC109 cells were grown in plates until 70-90% confluence. The sequence of the siRNA-LDHA was 5′- GACUGAUAAAGAUAAGGAATT-3′ and siRNA-GPR81 was 5′-GCAATTGTGTTCATCACATGC-3′ were purchased from GenePharma company (Shanghai, China). The cells were subjected to lip2000 (ThermoFisher, USA) and siRNA (3µM) treatment for 24 h, followed by treatment with BCT or LA for an additional 24 h before being harvested.

### Western blotting analysis

Western blotting analysis was used to perform the protein expression of relative genes. Briefly, the pretreated EC109 were lysed in RIPA lysis buffer containing protease inhibitor and phosphatase inhibitors cocktail (Jas enzyme, China). The supernatant was used to detect the concentration of protein by BCA protein assay Kit (Beyotime, China). Glut1, HK2, LDH, TLR4, p-Akt, HIF-1α, Wnt, β-catenin, N-cad, E-cad, Vamentin (Proteintech, China) primary antibodies were diluted 1000 times and β-actin diluted 5000 times were used in western blotting. The goat anti-rabbit or anti-mouse IgG secondary antibody was diluted by 1:5000. Subsequently, Omni-ECL™pico pight chemiluminescence kit (Jas enzyme, China) was used to analyze the protein brand under enhanced chemiluminescence system (Tanon, China). Band intensities from three biological experiments were quantified by densitometry using ImageJ software.

### Statistical analysis

SPSS 25.0 and R software were used to complete the statistical analysis. Differences among groups were compared with T test and Wilcoxon rank sum test. Univariate statistical analysis included t-test and multiple of variation analysis. Difference in bacteria among tumor and para-tumor tissue was detected by linear discriminant analysis, Kruskal Wallis test. The significance threshold was set at *P* < 0.01 (**) and 0.05 (*).

## Results

### Identified BCT as the virulence factor of F.p

The complete gene sequencing and protein sequence of F.p and F.n were obtained from the National Center for Biotechnology Information (NCBI) website https://www.ncbi.nlm.nih.gov/ and Uniprot http://www.uniprot.org/ respectively. Meanwhile, NCBI-protein BLAST https://www.ncbi.nlm.nih.gov/ used for sequence alignment. We founded that there is a gene of F.p which we called it BCT was similar to the FAP2 of F.n. The sequences were list in Table S1. Next, we will analyze the effect of BCT on EC109 cells. Therefore, the pET-28a prokaryotic expression vector of BCT was successfully constructed. The plasmid profile type was as Fig. S1A. As the Fig. S1B, 1, 2, 3, 4, 5, 6 represent respectively different optimum inducement time and concentration [control; 16℃, 12 h, 0.5mM; 30℃, 4 h, 0.5mM (supernatant)], [(control; 16℃, 12 h, 0.5mM; 30℃, 4 h, 0.5mM) (sediment)] after optimizing prokaryotic expression conditions, we determined the optimum inducement time and concentration of BCT was 16℃, 12 h and 0.5mM. Subsequently, the purified BCT protein was obtained with higher concentration and better purity resulting in a35 kDa band (Fig. S1C).

**Fig. 1:**
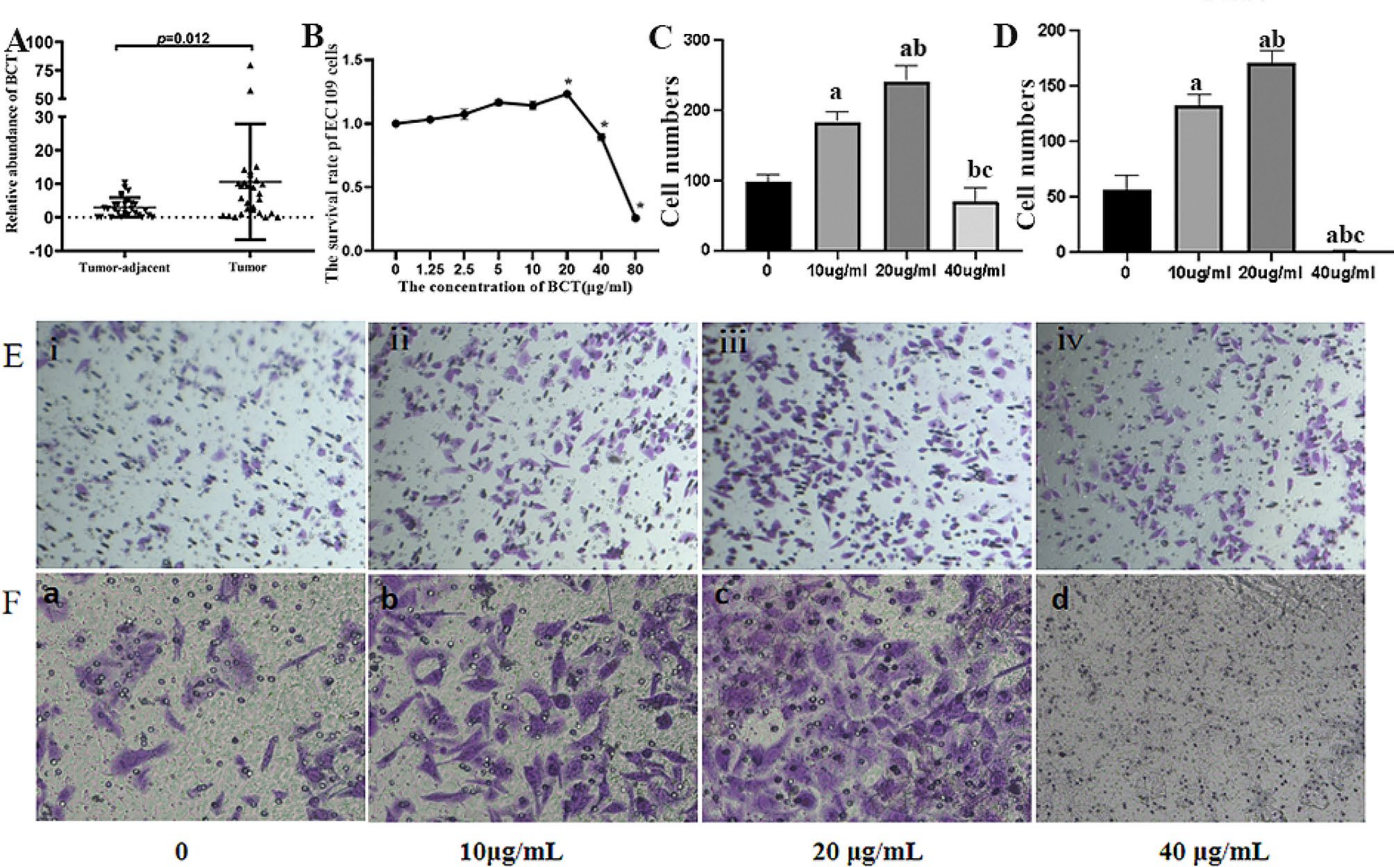
BCT advocates for the promotion of EC. (**A**) The expression of BCT in EC tumor tissue and para-tumor tissue. (**B**) The survival rate of EC109 after treated by different concentration BCT. (**C**) The number of cells for migration. (**D**) The number of cells for invasion. (**E**) The migration ability of EC109 in different concentration BCT, i, ii, iii, iv stands for control, 10 µg/mL, 20 µg/mL, 40 µg/mL respectively. (**F**) The invasion ability of EC109 in different concentration BCT, a, b, c, d stands for control, 10 µg/mL, 20 µg/mL, 40 µg/mL respectively. Results are representative of three independent experiments, and expressed as mean ± SEM. *, *p* < 0.05

**Fig. 2:**
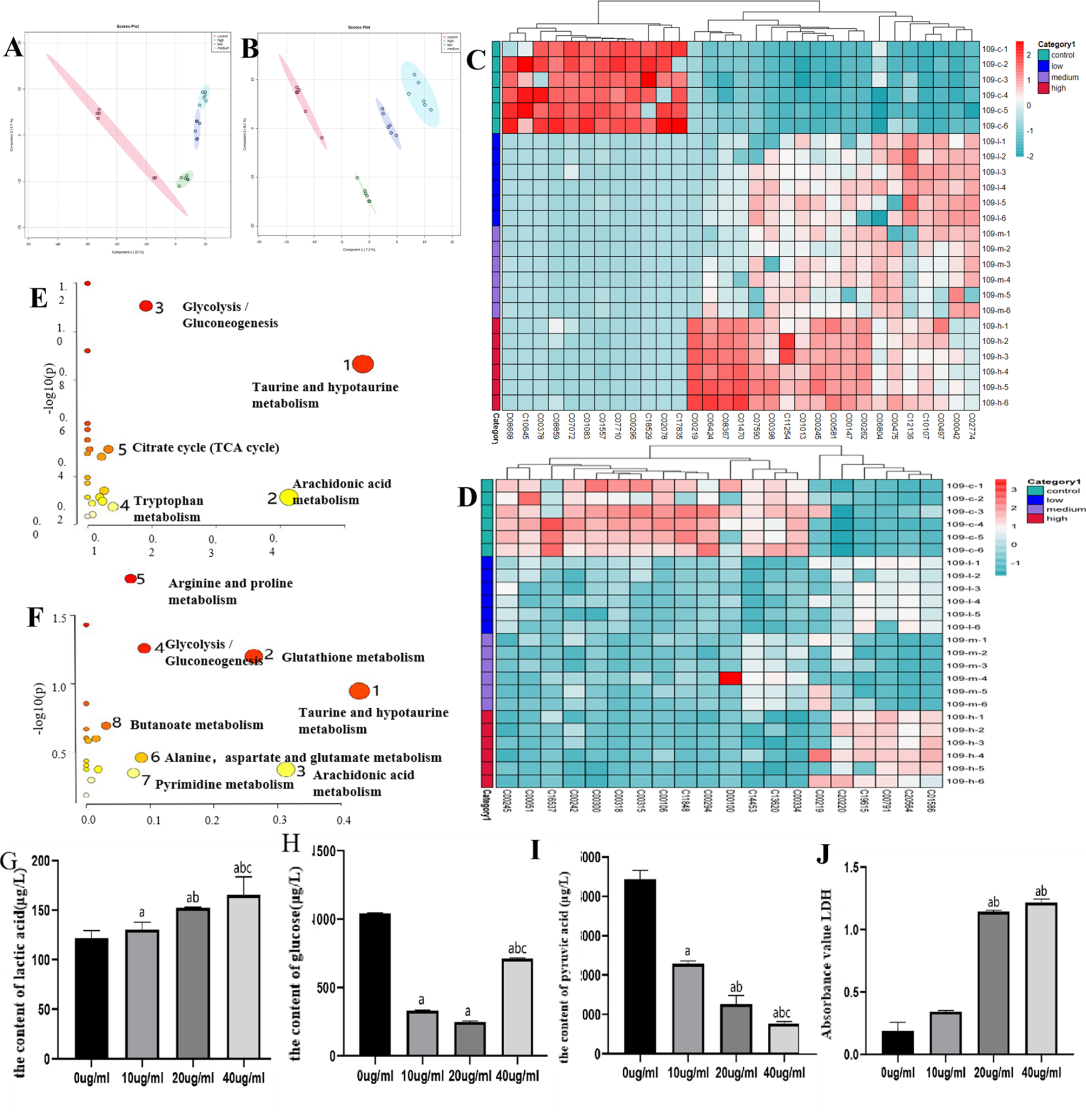
Metabolomic changes in EC109 cells following BCT treatment. (**A**) PCA analysis indicated that BCT lead to the difference between control and different BCT concentration in supernatant. (**B**) PCA analysis indicated that BCT lead to the difference between control and different BCT concentration in cells. (**C**) Differential metabolite in supernatant. (**D**) Differential metabolite in cells. (**E**) KEGG enriched metabolic pathways in supernatant. (**F**) KEGG enriched metabolic pathways in cells. (**G**) The content of glucose in EC109 after treated by BCT. (**H**) The content of LA in EC109 after treated by BCT. (**I**) The absorbance of LDH in EC109 after treated by BCT. (**J**) The content of pyruvic acid in EC109 after treated by BCT. Results are representative of three independent experiments, and expressed as mean ± SEM. *, *p* < 0.05

**Fig. 3:**
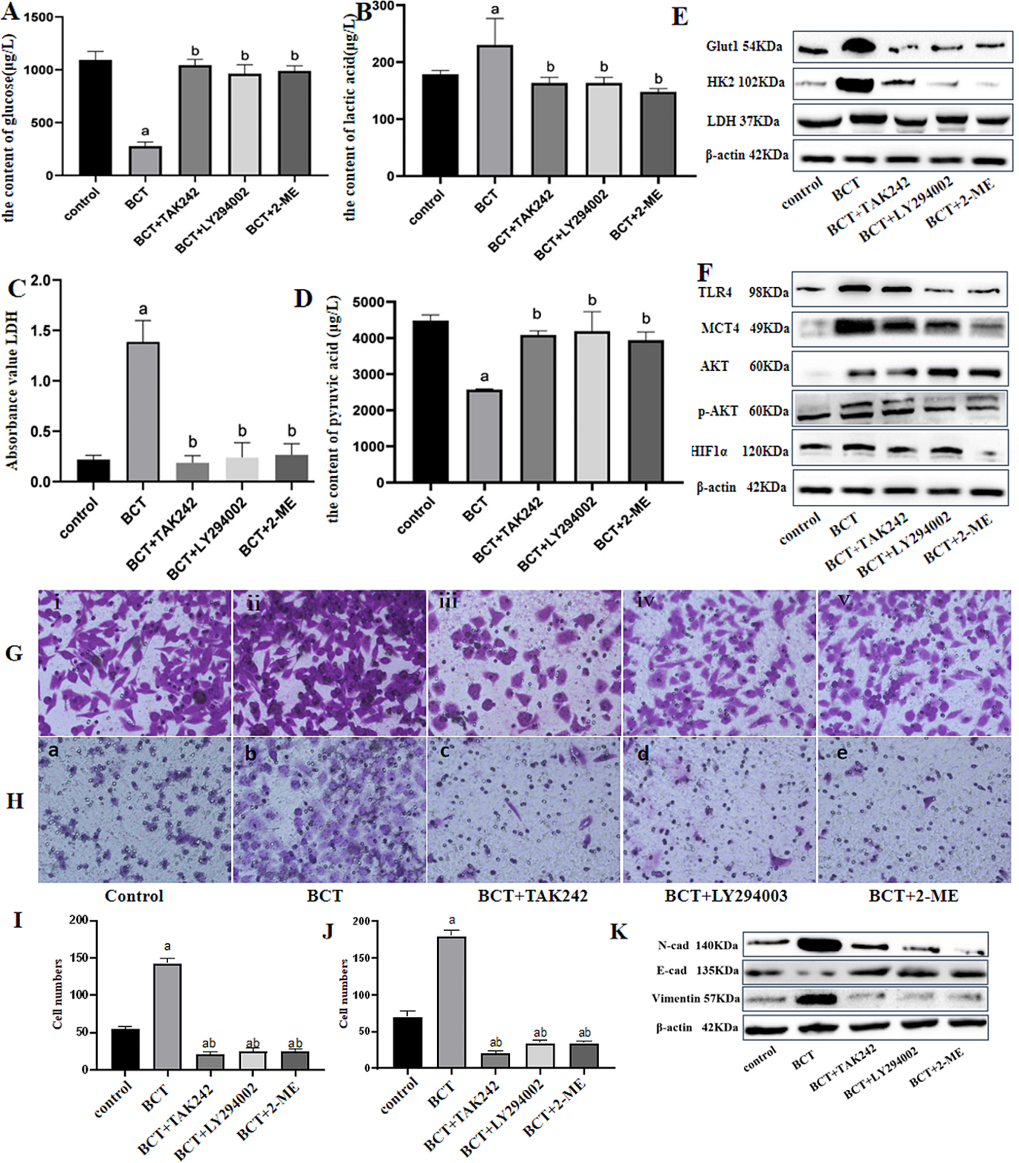
BCT promote EMT by modulating the glycolysis in EC109 cells through TLR4/Akt/ HIF-1α signaling pathway. (**A**) The content of glucose in EC109 after treated by BCT and inhibitor. (**B**) The content of LA in EC109 after treated by BCT and inhibitor. (**C**) The absorbance of LDH in EC109 after treated by BCT and inhibitor. (**D**) The content of pyruvic acid in EC109 after treated by BCT and inhibitor. (**E**) The protein expression band of Glut1, HK2, LDH which related to glycolysis. (**F**) The protein expression band of TLR4, p-Akt, HIF-1α, β-actin as internal reference gene. G.BCT promote the migration of EC109, i represent the control group, ii represent the BCT group, iii represent the BCT and TAK242 group, iv represent the BCT and LY294003 group, v represent the BCT and 2-ME group. H. BCT promote the invasion of EC109, a represent the control group, b represent the BCT group, c represent the BCT and TAK242 group, d represent the BCT and LY294003 group, e represent the BCT and 2-MR group. I. The number of cells for migration. J. The number of cells for invasion. K. The protein expression band of N-cad, E-cad, Vimentin which related to EMT. Results are representative of three independent experiments, and expressed as mean ± SEM. *a* < 0.05 was for comparison with the control group, *b* < 0.05 was for comparison with the BCT group

**Fig. 4:**
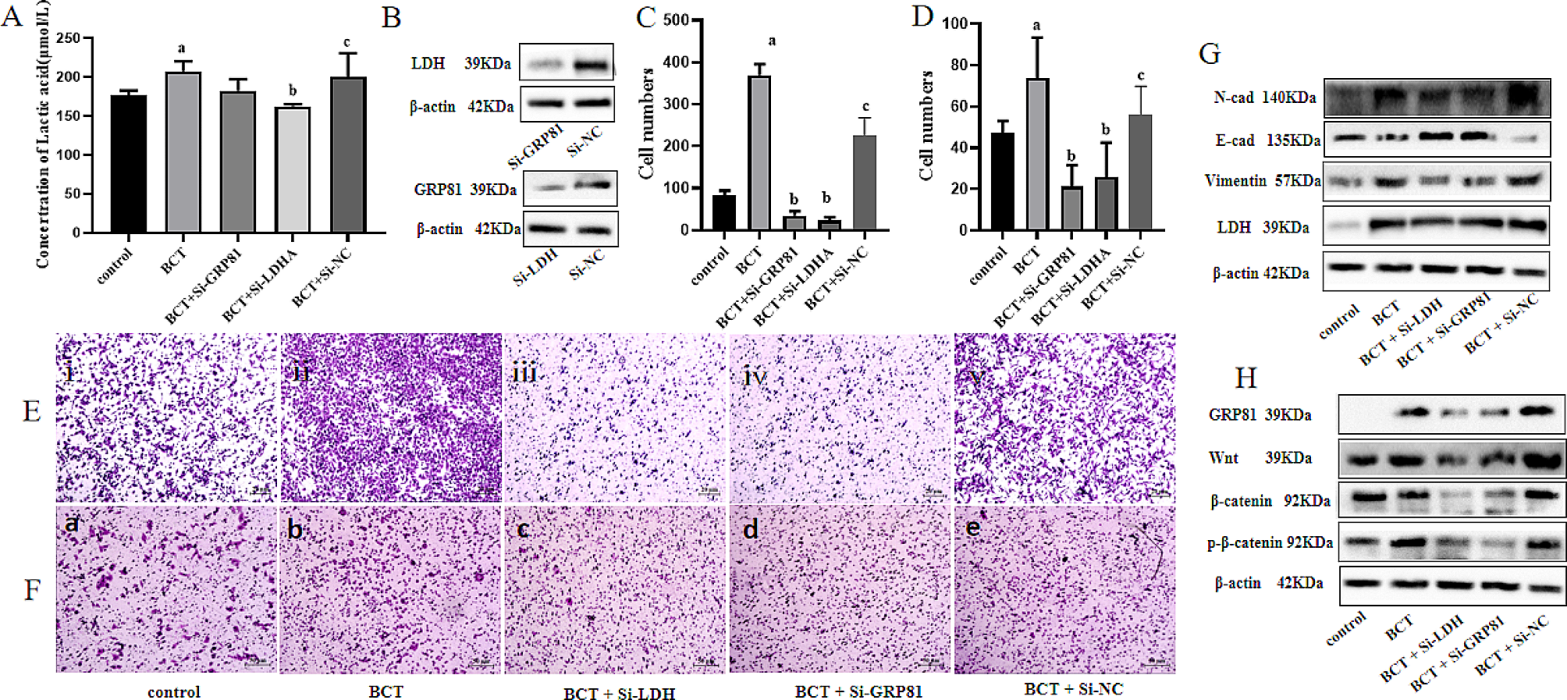
EC109 glycolysis and released LA promotes the EMT. (**A**) The concentration of LA after treared by BCT, Si-LDHA and Si-GPR81. (**B**) The interfering availability of Si-LDHA and Si-GPR81. (**C**) The number of cells for migration. (**D**) The number of cells for invasion. (**E**) The migration ability under different processing, i represent the control group, ii represent the BCT group, iii represent the Si-LDHA group, iv represent the Si-GPR81 group, v represent the Si-NC group. (**F**) The invasion ability under different processing, a represent the control group, b represent the BCT group, c represent the Si-LDHA group, d represent the Si-GPR81 group, e represent the Si-NC group. (**G**) The protein expression band of N-cad, E-cad, Vimentin which related to EMT. (**H**) The protein expression band of GPR81, Wnt, β-catenin. Results are representative of three independent experiments, and expressed as mean ± SEM. *a* < 0.05 was for comparison with the control group, *b* < 0.05 was for comparison with the BCT group. c < 0.05 was for comparison with the SiRNA

**Fig. 5:**
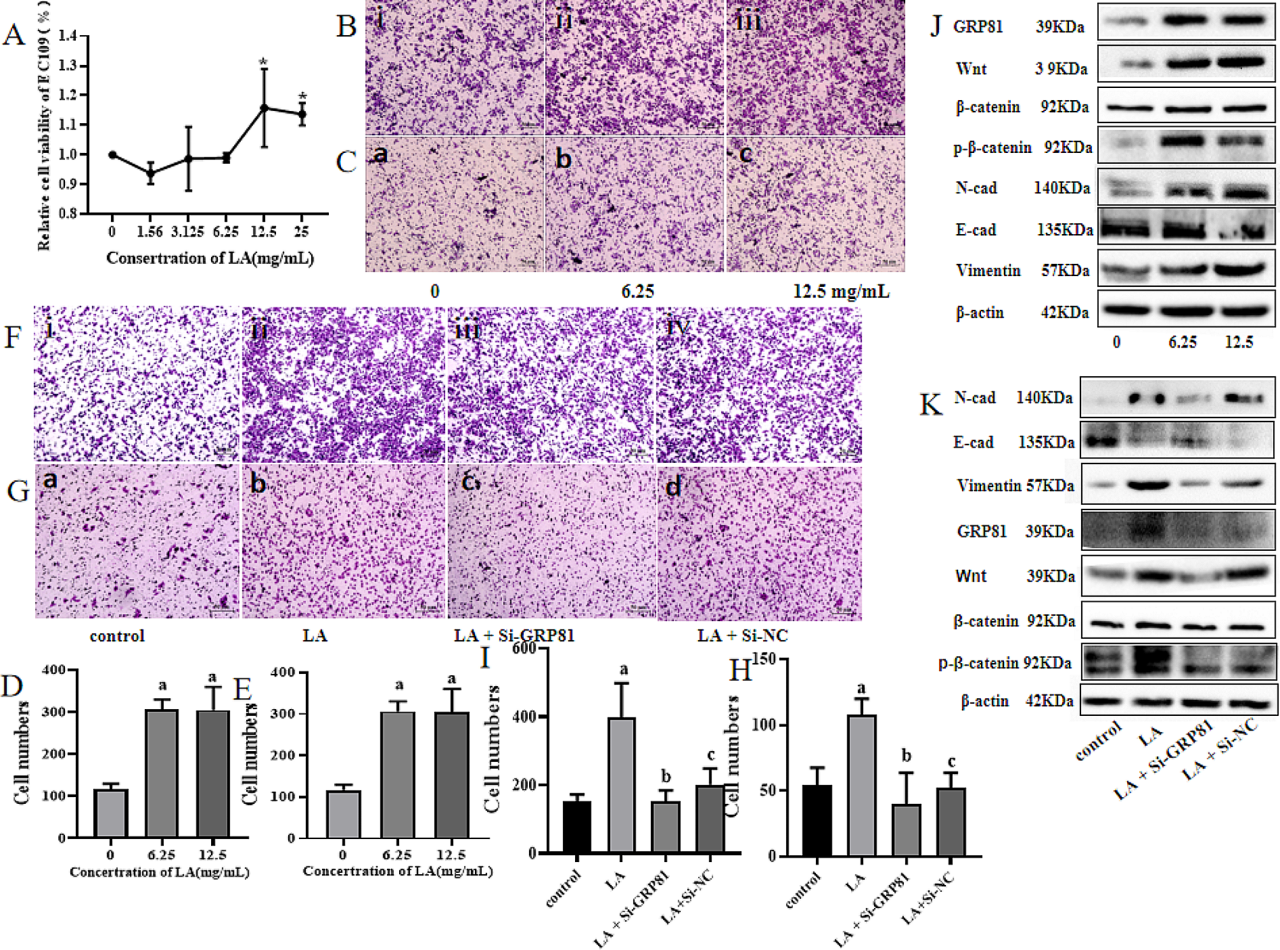
LA promotes the EMT of EC109 through GPR81/Wnt/β-catenin pathway. (**A**) The survival rate of EC109 after treated by different concentration LA. (**B**) Different concentration LA promote the migration of EC109, i, ii, iii represent the control, 6.25 mg/mL, 12.5 mg/mL. (**C**) Different concentration LA promote the invasion of EC109, a, b, c represent the control, 6.25 mg/mL, 12.5 mg/mL. (**D**) The number of cells for migration. (**E**) The number of cells for invasion. J. Western blotting was performed to detect the expression of related genes. (**F**) The migration in LA group and siGPR81 group, i, ii, iii, iv represent the control, LA, LA with siGPR81, LA with siNC. (**G**) The invasiveness in LA group and siGPR81 group, a, b, c, d represent the control, LA, LA with siGPR81, LA with siNC. (**H**) The number of cells for migration. (**I**) The number of cells for invasion. H. Western blotting was performed to detect the expression of related genes. *P* < 0.05

**Fig. 6:**
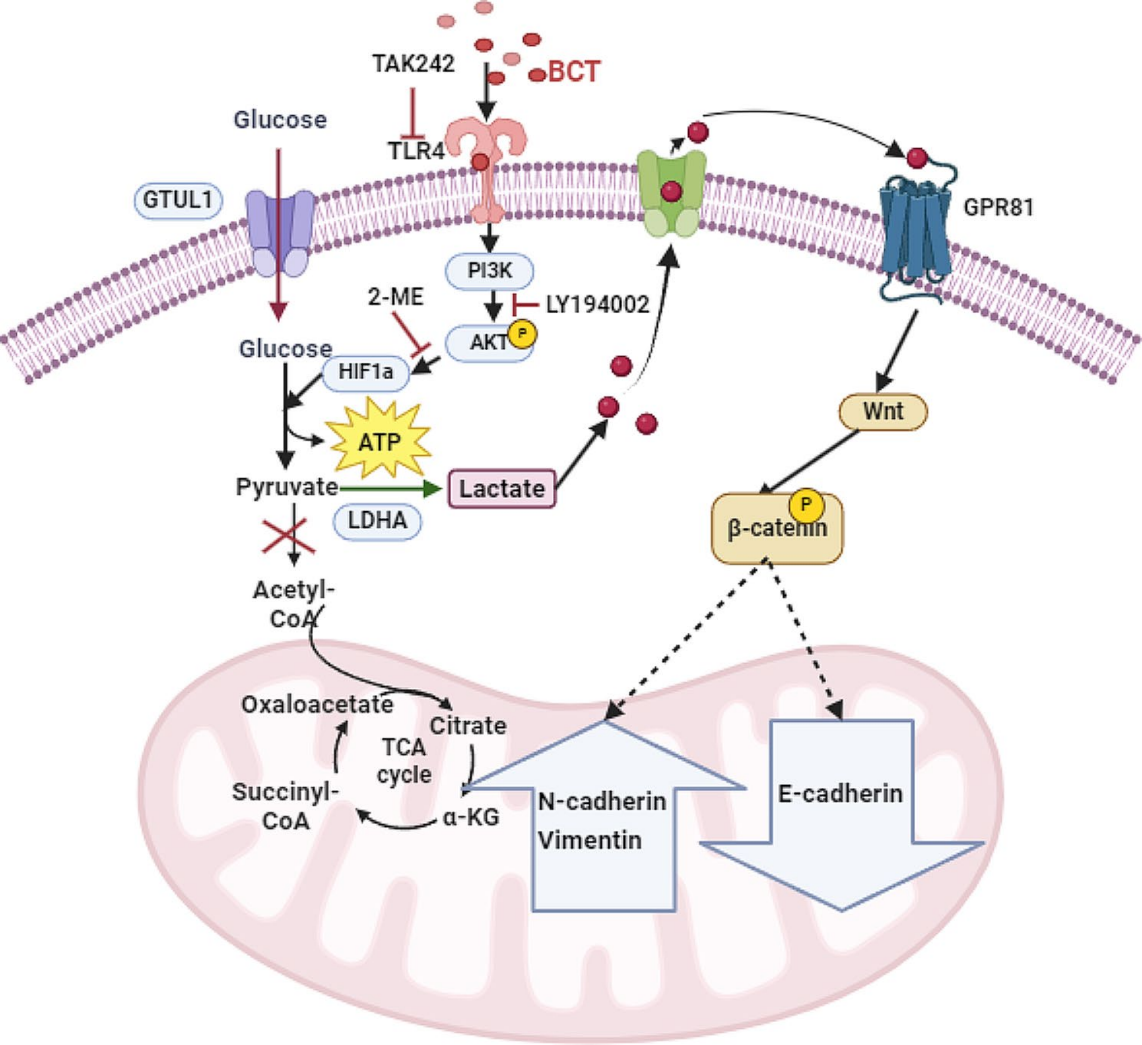
Schematic representation of BCT promote the EMT of esophageal cancer through glycolysis release lactic acid

### The abundance of BCT in ESCC and effect on EC109

In our previous study, we have observed a significant abundance of F.p in ESCC tissues compared with adjacent tissues. Additionally, using RT-QPCR analysis, we found that BCT was more frequently expressed in cancerous tissues of 33 ESCC patients (Fig. 1A). Hence, we thought that BCT may play a crucial role in the progression of ESCC. To validate this hypothesis, we conducted experiments using EC109 cells. CCK8 result showed that the BCT promote the survival of EC109 in the concentration of 20 µg/mL but inhibit grow of EC109 in 40 µg/mL and even higher concentration at 24 h (Fig. 1B). And the transwell assays demonstrated that BCT increase the migration (Fig. 1C**&E**) and invasion (Fig. 1D**&F**) of EC109 in 20 µg/mL but 40 µg/mL ). So, these results collectively indicate an abundant presence of BCT in tumor tissues and 20 µg/mL BCT promote the proliferation, invasion and migration of EC109 cells.

### BCT induces metabolic remodeling in EC109 cells

To investigate the effect of BCT on EC109 cells metabolism, we analyzed the cell supernatant and precipitate of EC109 treated by different concentration BCT (10 µg/mL, 20 µg/mL, 40 µg/mL) using UPLC-MS/MS. PLS-DA analysis indicated that the metabolic profile including the type or level of metabolites of BCT co-culture cells had significantly changed (Fig. 2A**&B**). We also compared the metabolites in different groups and found 49 (Control vs. 10 µg/mL), 46 (Control vs. 20, 10 µg/mL), 62 (Control vs. 40, 10 µg/mL) differential metabolites respectively. Among these, we screened out a set of 32 metabolites that were consistently altered across all treatment groups, and the hetmap was as Fig. 2C**&D**. Additionally, 32 metabolites were analyzed for enrichment by KEGG. As the Fig. 2E**&F**, the main metabolic pathways in cell supernatant were taurine and hypotaurine metabolism, arachidonic acid metabolism, glycolysis/gluconeogenesis, tryptophan metabolism, citrate cycle. The main metabolic pathways in cells were taurine and hypotaurine metabolism, glutathione metabolism, arachidonic acid metabolism, glycolysis/gluconeogenesis, arginine and proline metabolism, alanine, aspartate and glutamate metabolism, pyrimidine metabolism, butanoate metabolism. Hence, we detected the content of key products of glycolysis process such as glucose, LA, LDH and pyruvic acid. These results indicated BCT decreased the glucose (Fig. 2G) and pyruvic acid (Fig. 2H) but increased LA (Fig. 2I) and LDH (Fig. 2J). These findings suggest that BCT can remodel EC109 cell metabolism primarily through its effects on glycolytic processes.

### BCT promote EMT by modulating the glycolysis in EC109 cells through TLR4/Akt/ HIF-1α signaling pathway

We further investigated the underlying mechanism by which BCT promotes glycolysis in EC109 cells. Previous studies have implicated TLR4 plays a key role in glucolipid metabolism, which is linked to the development of chronic inflammation and the progression of cancer [42]. In this study, we explored the role of TLR4 and PI3K signal pathway in regulating glycolysis. The WB results showed that BCT increased the expression of TLR4, p-Akt, HIF-1α. However, after treated by different inhibitor (TAK242 for TLR4, LY294002 for AKT, 2-ME for HIF-1α), the glucose (Fig. 3A) and pyruvic acid (Fig. 3D) was increased but LA (Fig. 3B) and LDH (Fig. 3C) was decreased as well as the expression of Glut1, HK2, LDH were all decreased compared with the BCT treated group (Fig. 3E**)**. Meanwhile, after treated by different inhibitors, the expression of TLR4, p-Akt, HIF-1α were also changed (Fig. 3F**)**. Collectively, our findings underscore that BCT enhances glycolytic metabolism in EC109 cells through modulation of the TLR4 /Akt /HIF-1α signaling pathway.

In cancers, EMT is considered to be a driving force to promote tumor invasion and metastasis [43]. Therefore, we investigated the potential of BCT to induce EMT in EC109 cells and its association with glycolysis. We initially demonstrated that BCT enhances the invasiveness and migratory capacity of EC109 cells. However, inhibition of TLR4/Akt/HIF-1α signaling pathway reduce the glycolysis process resulted in decreased release of LA, and the invasiveness and migratory capacity of EC109 cells was decreased (Fig. 3G**&H&I&J**). Meanwhile, we conducted an analysis on the expression of N-cadherin (N-cad), E-cadherin (E-cad), Vimentin, and observed that BCT treatment resulted in an upregulation of N-cad and Vimentin expression but E-cad (Fig. 3K). Importantly, these changes were counteracted by the inhibitors targeting TLR4/Akt/HIF-1α (Fig. 3K). The findings suggest that BCT may enhance EMT in EC109 cells, potentially through the activation of the TLR4/Akt/HIF-1α signaling pathway, which is associated with glycolysis progression.

### BCT induced EC109 glycolysis and released LA promotes the EMT by GPR81/Wnt/β-catenin pathway

LA is a classical byproduct of glycolysis [44]. Recent years, its invasive and migratory potential of cancer was significantly enhanced [41]. In order to elucidate whether the role of BCT in promoting EMT of EC109 cells was related to LA accumulation. Si-LDHA was used to inhibit the LA production, and the result found that Si-LDHA reduced the production of LA (**Fig. 4A**) and decreased the invasive and migratory of EC109 treated by BCT (**Fig. 4E-iii&F-c**). The Si-LDHA treatment altered the BCT-induced upregulation of N-cadherin and vimentin, as well as downregulation of E-cadherin (**Fig. 4G**). Hence, we thought that BCT promote the EMT of EC109 by releasing LA. Meanwhile, we also explored the mechanism of LA promote EMT. There were evidences indicated that LA could combined with GPR81 and regulated the progression of cancers [45, 46]. Our findings demonstrate that Si-GPR81 significantly attenuates the invasive and migratory capabilities of EC109 cells compared to those treated with BCT (**Fig. 4E-iv&F-d**). The expression of EMT-related genes was also altered by Si-GPR81. Previous research has demonstrated the regulatory role of GPR81 in the Wnt signaling pathway, which plays a crucial function in EMT [47, 48]. We evaluated the gene expression of GPR81/Wnt/β-catenin by WB and found that BCT significantly increased the gene expression of GPR81, Wnt and p-β-catenin, while Si-GPR81 reduced the gene expression levels of GPR81, Wnt and p-β-catenin (Fig. 4H).

Meanwhile, in order to further investigate the role of LA in EC, exogenous LA was administered to EC109 cells and its potential for promoting cancer was evaluated. CCK8 used to detect the role of LA in EC109 and the result showed LA in 12.5 mg/mL and 25 mg/mL promote the proliferation of EC109 (Fig. 5A). And as the increasing of concentration of LA, the migration and invasion ability were all increased (Fig. 5B**&C**). Similarly, exogenous LA also increased the expression of GPR81, Wnt, β-catein, p-β-catein, N-cad, Vimentin but E-cad (Fig. 5J). However, under the role of Si-GPR81, the migration and invasion ability were all reduced (Fig. 5F**&G**), as well as the protein level of GPR81, Wnt, β-catein, p-β-catein, N-cad, Vimentin but E-cad (Fig. 5K). Collectively, these findings suggest thatlactate released due to aerobic glycolysis induced by BCT treatment promotes EMT via activation of the GPR81/Wnt/β-catenin signaling pathway.

## Discussions

Enhanced evidence suggests the pivotal role and significance of microbiota in cancer development. Helicobacter pylori, Fusobacterium nucleatum, Escherichia coli, Bacteroides fragilis and Porphyromonas gingivalis had been identified as potential carcinogenic effects [49]. As an important microbe infiltrating in esophageal cancer microenvironment, understanding the role and mechanism of FP in esophageal cancer may provide theoretical support for the prevention and treatment of esophageal cancer by targeting FP. Hences, this study reported a virulence factor BCT of F.p in promoting of ESCC. We founded that BCT was high abundance in ESCC tissues compraed para-tumor tissues. By using metabolomics and microbiomics analyses of EC109 treated by recombinant protein BCT, we found the BCT modulated glycolysis. The mechanism investigation further revealed BCT promote glycolysis was regulated by TLR4/Akt/HIF-1α signaling pathway. We then discovered that the BCT enhances the EMT-mediated metastasis of EC109 cells. The EMT-mediated metastasis is associated with an increase in glycolysis caused by BCT, which promotes the production of LA. Consequently, this leads to the induction of EMT in EC109 cells through the regulated GPR81/Wnt/β-catenin pathway. These findings collectively suggested that the BCT could serve as a potential therapeutical target for F.p infected EC patients.

Pathogenic bacteria have evolved diverse strategies to target the host, with one of the most significant mechanisms being the manipulation of host cell signaling pathways through virulence proteins, thereby eliciting various biological effects. For instance, cytotoxin-associated gene A (CagA), a major virulence factor in Helicobacter pylori, promotes the progression of gastric cancer through the miR-155-5p/SMAD2/SP1 axis [50]. Fap2 serves as the principal virulence factor of Fusobacterium and is implicated in the pathogenesis of various types of cancer [51–53]. By comparing sequences from F.p and F.n using NCBI database analysis, we discovered a BCT sequence in F.p that shares similarity with Fap2. Consequently, we designed primer for BCT and analyzed its abundance in both EC tissues and para-tumor tissues. Our results revealed higher levels of BCT expression in EC tissues compared to para-tumor tissues. Subsequently, the effect of BCT on EC cells was studied by EC109 cell model. We found that a concentration of 20 µg/mL BCT increased the survival rate, invasion, and migration of EC109. Therefore, we conclude that BCT derived from F.p plays a crucial role in influencing ECdevelopment.

The modulation of metabolic levels by virulence factors is a crucial determinant in the progression of tumors. For instance, the W83 membrane component of Porphyromonas gingivalis induces significant metabolic gene alterations in Oral Squamous Carcinoma Cells [42]. In this study, we first explored the BCT promote the metastasis of EC109 whether related to metabolic reprogramming and the result found that BCT alters the state of metabolism, specifically by increasing glycolytic activity in EC109 cells. Furthermore, we analyzed the metabolites involved in glycolysis and observed an increase in glucose and pyruvic acid levels, accompanied by a decrease in lactic acid and LDH levels. Notably, Glut1, a key glucose transporter crucial for regulating tumor energy metabolism [54], along with Hexokinase (HK), one of the three key regulatory enzymes initiating aerobic glycolysis by phosphorylating glucose were upregulated after BCT treatment [55], Additionally, LDH, which catalyzes the final step of glycolysis converting pyruvate into lactate, showed increased expression following BCT treatment [56]. Our results indicate an upregulation in the expression of proteins Glut1, HK2, and LDH of EC109 after treated by BCT, which are involved in regulating the production of related metabolites.

Meanwhile, research has demonstrated that F.n induces an inflammatory response mediated by TLR4 stimulation [57]. Additionally, TLR4 is known to play a crucial role in the regulation of glycolysis and pyruvate oxidation decarboxylation, with its expression being upregulated in various types of cancer [58–60]. In our study, we observed an upregulation of TLR4 expression in EC109 cells following treatment with BCT, while it was downregulated by the inhibitor (TAK242) [61]. Previous studies have demonstrated that the PI3K/Akt pathway is intricately regulated and plays a pivotal role in modulating glucose metabolism in cancer cells. Additionally, this pathway also governs the expression of HIF-1α [62–64]. It is noteworthy that the PI3K/Akt signaling pathway can be modulated by TLR4 [19]. Hence, we analyzed the role of TLR4/Akt /HIF-1α pathway in regulating glucose metabolism of EC109 treated by BCT. Our results showed that BCT activated the TLR4/Akt/HIF-1α inhibited TLR4. Conversely, the TAK242 (inhibitor of TLR4) inhibited the TLR4/Akt/HIF-1α, LY294003 (inhibitor of Akt) inhibited the Akt/HIF-1α [65], 2-ME (inhibitor of HIF-1α) inhibited the HIF-1α [66]. Furthermore, metabolisms changes were observed after treatment with TAK242, LY294003, 2-ME compared to BCT treatment. Based on these findings, we propose that the TLR4/Akt/ HIF-1α pathway participates in regulating glycolytic metabolites in EC109 cells following treatment with BCT.

Our study also observed BCT promote the EMT-metastatic of EC109. Previous studies have indicated that the HIF-1α in the tumor microenvironment promotes the expression of VEGF, HGF, met proton genes, and induces the degradation of extracellular matrix, which is involved in the mechanism of cancer cell metastasis known as EMT [67]. HIF-1α serves as a pivotal regulatory factor in the context of LA with cancers. Additionally, our study demonstrated that BCT inhibits the expression of HIF-1α as well as reduces the production of LA. This observation inspired us to hypothesize that whether the BCT contributed to EMT of EC109 was related to LA. Our result observed that LA supplement contributed to EMT of EC109 but the EMT-metastatic was reduced after knockdown the LDHA. Meanwhile, LA participated in the compound of collagen by TGF-β/Smad, Wnt/β-catenin, IL-6/STAT3 and promote EMT [40]. G protein-coupled receptor 81 (GPR81) as the receptor of LA could activate the Wnt pathway and regulate the proliferation and migration of cancer [68, 69]. Our results found that exogenous LA activated GPR81/Wnt/β-catenin and promote EMT but inhibited by Si-GPR81. Importantly, our findings demonstrate that exogenous LA activates GPR81/Wnt/β-catenin signaling pathway leading to enhanced EMT but this effect is inhibited by Si-GPR81.

In summary, we have identified the BCT protein as a crucial virulence factor of F.p and confirmed its role in EC pathogenesis. We have demonstrated that BCT alters the metabolic profile of ECs and enhances glycolysis and release LA, which is regulated by the TLR4/Akt/HIF-1α signaling pathway. Meanwhile, LA promotes EMT and enhances migratory and invasive capabilities of EC109 through GPR81/Wnt/β-catenin signaling pathway (Fig. 6**)**. These findings highlight the significance of BCT as a key virulence protein for F.p in promoting EC progression, with metabolic reprogramming induced by BCT being a critical biological process in this context. Hences, blocking the translocation from oral cavity to esophagus, such as improving oral hygiene to reduce might become the reliable interventions to prevent esophageal cancer. Meanwhile, our study suggests that targeting BCT could be explored as a novel therapeutic approach for personalized treatment for the subset of treated recalcitrant EC patients carrying the F.P.

### Electronic supplementary material

Below is the link to the electronic supplementary material.

Supplementary Material 1

Supplementary Material 2

## Abbreviations

Akt: Serine/Threonine Kinase 1
EC: Esophageal cancer
ELISA: Enzyme-linked immunosorbent assay
EMT: Epithelal-mesenchymal transition
Fap2: Fibroblast activation protein 2
F.n: Fusobacterium nucleatum
F.p: Fusobacterium periodonticum
GLUT1: Glucose transporter 1
GPR81: G protein-coupled receptor 8
HIF-1α: Hypoxia Inducible Factor 1 Subunit Alpha
HK2: Hexokinase 2
LA: Lactic acid
LDHA: Lactic dehydrogenase A
NCBI: National Center of Biotechnology Information
PMSF: Phenylmethylsulfonyl fluoride
TME: Tumor microenvironment
TLR4: Toll-like receptor 4
CagA: Cytotoxin-associated gene A
