# Supplementary material for: Fusobacterium periodonticum BCT protein targeting glucose metabolism to promote the epithelial-mesenchymal transition of esophageal cancer cells by lactic acid
Source: J Transl Med. 2024 Apr 30;22:401. doi: 10.1186/s12967-024-05157-z (PMC11061911; doi:10.1186/s12967-024-05157-z)
Supplement: Supplementary file 1 — Supplementary Material 1 [file 12967_2024_5157_MOESM1_ESM.docx]

Table S1 Nucleotide sequence and Amino acid sequence of virulence factor Fap2 of Fusobacterium nucleatum and Fusobacterium periodonticum

| Name | Sequence |
| --- | --- |
| Nucleotide sequence of virulence factor Fap2 of Fusobacterium nucleatum | ATGGAAGCACCAGAAATAAAACCAGAGCCAAATAAACCTATTGAGGCTCCAAAAATAACATTACCTACAATAAATAAAGTAATAATAGAGGAATTAAATATTACACCTCCAGCAGCATTAAATGTACCAGGGACACCTAATATTAATATAACAATAAATGCACCAAATGCACCAACACCACCAAGTGTAAGTGTAAATGTTAGTGAACCAAGTGCACCAAATGCACCAAATATAAATATTCCTATAACTCCACCAGAGATAAAGGCATTAAATATAGAAACACCACCAACAGTGAATGTTGCACCACCGACAGTAGCAGCAATAAATCCTGTTGCATTTTCTGTTGCTCCTACAATAGATTCAAAACAGTATAAATTTGGTGGAGCTAATATCAATAATGGCTTGAATGGTTTACCTTCTACAATAGATGTTCAAAGTAATTCTAATACAAATAGAAACTATGTAACAATAAATGCAAGTGGTTCATCTGTTCCTACTTCTGTTTTACTACCTGATAAAAAAACTATTAATGTTTCTGTTAATAATAACAGAGCCTTAGT  TGTGGATGAGGCAAGAGAAAATTTTGATTTTCAGATGAAAGGGACAATTAATTTATATGGCAATCAAAATATGGGTATAGATTTACAAGGAACTCATATAGGTGGATCTGCAAAAGGAAGTGCAACAAATCCTGCAATATCTACTATTAGAAACGAAGGAGTAATTACAGGGCATGCTACAAATAAGTATGATAACAATAAAGCTACTAAAGAACAAGTTGCCTTTGGTTTTAGTAATGCAGATGCTTCAAGCAATGCAACAATGACTCATATGATTAATAATAATGAGATAACTTTAAATGCTCCTTCAAGTGCAGGAATTCAATTGAAACCTGAAGATCCTTTTTATTGGGATCCAAATTGGGGAACTTTATCAAGTGATAATAAAATGATTATAAATGGTCTTAGTGGTTCTAGAAATGCTAATAATTTTGGTAGAGTTTTAATGAAGGCTGACAATAGAAAAGATATTAATTTGAATGGTAGTGGAAGTTTTGGAATGATTACTGTTTTCAACCCAGGAGTTATTGAACTTAATACAATAAGTGTCAATAGAACTACTTATCCCACAGGAAGCTATAATTTAAGAGCTCAAAGAAATATAGGGACAAAAGTTTTACCAGGTGGGGAAATAGGTCGTTCTGCTTTATCTGATTCTAAGTATACAAGTGGAGTTTATAACTCAGGAAATATTAATATAAATGGTGATGAAAGTGTTGGGGTTGGTATCCTTCATGAAATACAAGAAGTAAAAATAGGAGGAACTATAAATATTGGAGTGGAAAGTGTTAGTCAAACTACTGGAATTTCTGATACAAAATCTACAACTGGAAATGACCAAACAACCTTAGTTAACAATGCAGTAGGAGTATTTTCTGGTGTTCCAACTCTTCCAGTAAAAAATAATGAATATGATACTATGGGAAATAAAAATACTACTGGAAATATTATTGGAACTGAAACTAGTGAAGTTGATGGGACAATTAATATTGGAAGACATTCAAAAGAAAGTATAGGGCTTTTAGTTGGAGATAGTGGAGAAACTTTAAATAATGGAACATTAAATGGAACACCAAATCAAGCTAGGATATTAAAAAGATCTGGTTCTATTACTTATAAATCTTCTGCTAATAATAAGTTAAATATCAATGGAAATGCAAATTATGGTTTTGTAGTTAAAAGTGATTCTAATAAATCAGTATTTGGTTCTGCATTAGATGACTTACAACAAAGTGTGGATAAAACAAATTATGGTATAGGGATAAACAAAGGAAATATTGATATTATAGGAATTAATTCTATAGGTTTTGTACTTTTAAAAGGTGGAAATTCTAAGAATACAGGAAATATAACTGTAAATGAAAGTGAAGATTTTTGGTATCCTATTGTACCACCTACACCTAATTATGATTATAGACAAAATTCAATAGGTTTTTATGGAGAACAAGATAATTTTACTAATGAAGGAACAATAAGTGTTAATACTCCAAATAGAAGTGGAAATAAAGCTGTTCTTTTAAAAGGAAATAGCAATGGAATTACTTTTAATAATACAGGAGATGTTTCAGTAAAGGGTAGAAATAATATTGCGATTTATGCAGAAGGAAAGTACACTTTCAATCATGAAAAAAATGCTGCTGGAACTAATAAAATTAGTGTGGGTTCAAATTCAATAGGAATTTATGTGAAAGATAATACAGGAACTGTAAATATAAAAGCTCCTATTGAGTTAGCAGATAGTTCTAATGGAACAACTATTGGAGTTTATTCAGATGGAAATGCTCATATTAATTTTGAAAATGGATCAAAGTTAACAATAGGGAAAAAAGCTATTGGATTATTTTCACAAAGTACAACTAATTTTAAAAATACTTTCAAATTTAATAATACAGCAGGGAATGAATTAAATGTAAGTTTAGATGAAAACTCAGCTTTTGCATTTTTTAATGGAAGTGGAAGTACAGATATAGCAGAAGTGCTTAATAAAAATATAAAATTTACTGCTATGAAAAAAGGAGCTACTTTTGCTTATGTTAAAGGAGGTTCAATTGTGACTTTAAGTCATGATTTTGATACAACAAATACAGCAAAAGTTGTTGTAGCTCCAGAAAGTGGAACATCAGTCTTAGTTGCTAGTGATGGTTCAACGGTACAAGTAGATGCAAATAAAAAATTAACAACTAATACTAATGTTGGTTTGGTTGCAACTAATGGAAGTTCTTCAAATAAATCTAAGGCAATAAATAAGGGTACTATTATTTCAAAAGTAAATGGAGGAGTTGGACTTTATGCAAATAACAATAGTGAGGCAACAAATGATATAGCTGGAAAAATCACTATGGAAAATAAAGGCTCAGCAGCTATTTTAGGAGAAAATAATTCTATCCTTACAAATAAAAAAGATATTGAATTAAAAGAAGAAGAATCAGCAGGTATATATGCTAAAGATTCACTTGCAACTAATAGTGTGACAAGTGGTTCACCAGCAGTAAAAGCTAAAATTAGTTTAGAAAAAACGAAATCAGCAGGTATGTATTCTATATTAACTAATTTAGCAGATGGAGATAAAAAAATACTAAACCAGGCAAATGGAGCCGATAAAGCAAGTATTGAAATAGGAGCTAATGCAACATCATCAGCAGGTATGTTTGGAAAAGTTGAATCAAATGCAACAAAAGTATTAACATTAGAAAACGCAGGTAATATTCAAGTAGGAGCAACATCATCAGTAGGTATGTATGCACAAAATGAAACAGTAGATGAAGATAAATTACTTGTAAATAATACAGGACTTATTAATGTAACAAAAGAAAGTTCAGTAGGTATTAATGCAAGTAAAAGAGCTACTATAACTAACTCAGGAGTAACAGCTGATGGGAATGGTATAGTTTTATCTGCTAAAAAAACAGCTGGAATTATAGGAAATAATAACTCAGTTGTTACTAATACAGGAGATATAACTTTAAATAGTACAACACAACCAACTGATTCATCAGAAGGGTTGGTAGGAATATCAGTAGATAAAACTTCTACTGCTTTAAATAATGGAACAATAACTGTTAGTAATGATTATAGTACAGGAATTAGTGGAAAAGGTGGAAGAATCACTAATAATAATAAAATTATATTAAATAATATAAATAGTGTAGGAATTTCTTCTACCGATGGTTCAGTACTTAATAGTGCTGATACAAATAATCTTATTGAAGTAAAAAATTCTAATTCAGTGGGTATTTTTGCAAAGTTAATTGAAAATGTACCTTTTTCTCCATTCCAAGCTATATTAAATAAAGGAACAATAACTTTAAATGGTGGAAATTCTAAATCATCATCAGTAGGAATATATAGTTTAATTGAGGATAAAGCTGTTCAAAATAAATCGACACAACAAATAGCAAATACAGGTACAATAAATGTAAGTACAAAAGGTTCAGCAGGTATCTATGTGAAAGATAACAGAACATCAGGTACTGATGTTATTTCATTTATAAGAAATGAAAAAGATATAAATGTAGCAGAAGAGTCATCAGCAGGAATTATTGGAGAATATTCACAAATAACTAATGGAAATCCACAAAATACAAATGGCAAAATAGAACTTTCAGCCAAGAAAACAGCTGGAATTATTGCTAATAAAAAATCAGAAGTAATAAACTATGGAAAAATAGAAACATTAAGTTCTGTTACTATTAACAATAAAACTGATGCATTAGTAGGAATTTCAGTAAATAATTCTAAGGTTATTAATAAAAATAAAGAAGATTCAAATGGAATGATAGGAGCTATCACTTTAAATACAGCATATTCTACTGGTATTTATGGTAAAAATGAATCTACTTTAATTAATGAAGGAAAGATTACAGCTAATAAAACGGACTCTATTGGAATGGCAGGAGAAAATTCTTCTTTAACAAATAAAAATGAAATTCTAATAAATGAAGAAAAATCAGTAGGAATGTTTGGAACTACTGTTGGTAATAAAAATACCGTTCTAATTAATGAACAAGATGATATAAAGAGTTTAACTGGAAAAATTATAACTAAGAAAAAAGAATCAGTAGGAATGTTTGTTAGTACAAGTCAAGCTTCTGCTATAAATAGAGGAACAATTCTTATAGAAGAAGAAAAGTCAGCAGCTATGTATGGAGATGAAGCAAATATTGAAAATAGTGGAACTAGTACAAATTTAATTGCTTCTATAACAACTAAAAAAGATGAATCGGCTGGAATTTATGCTAAAAATAGTAATGCTACAAACAAGAAGAAGATGACTGTTGAAGGGAAAAAATCAGCAGCTATATTAGTAGAAATGACAAAAGATAAAAATATATCTGGAATTAATGATATAGCAGATAATAGTTCAGATATACTAAATGGACATATCGAAGTAAAGAATCAAGAATCAGCAGCTATGTATGGAAAAATTGATTCAAAAGTTTCAGTAGCTACTTCAACATTGACATTAATTAATAAAAAAGCTATTGATATAGATGCTAAATCATCAGTAGGAATGATGTTAATAAATGATTCAAATTCTGTTAATAAAACTAATGTAAAAGCTGAAAATAGTGGAGTTATTACTTTAAAAAATGCTACAAATAATACAGGGAATATAGGTATATTAGCTAAGAAAAACTCTACTGGAATTAACAAAGATAATGGAATTATTAATGTTAATAGTAAAAAATCAATAGGAATGTTAGCTAAGGAAGGTTCAAATGTAGAAAATAATTCTAATCTTCCACCTAATCCACTTGTAGTTGGTAAGGAATATGGTATTAATCTAAATGAAGAGGAAAGCATAGGAATGCTAGCAGAGGGTCAAAATTCTGCAAGTCAAGTTTCTACTGCTATAAATAATGCTAAAATATCTGTTACAGCAGCAGGTAAAAAGTCAATAGGAATGTTAGCTCAAAATGAAGGGGATGTAAAAAATAACAAAGAAATAGAAGTATTTGCTGAAAAAGGTGTAGGAATTTTTGTTTCTGATACAGGAACTGGTGAAAATACTAATCCTAATGGAAAAATTACTTTAATGAATAAAGAAGCAGTTGGAATTTTTGCTAAAAATAATGGAGATATATACACTGCCAAGAATTCTGGAAAAGTTATTTTAGGATCTGCTGATGGAACAACAACTCATGAATCTTTAATAGGTATGTTTGCACAAGCTGAAGCTGGGAAAAAATCTAGTATAAAGAACATAGATACTATTGATGTTAATACAAAAAAATCAGTAGGAATGTATGCAAAAAATGATGCTGCAAATGTAGATGATGTTGATTTATATAATGCAGGAACTATCAATGTAAACAATAAAGGTTCAGCAGGAATTTATGCACCTAAAGCTAATATTTCAAAAGTAGGAAATATCAAATTAAAAAATACAACTGATACAGATGGTGCTTCTGCTGTGTATATTTCACAAGGAGGAAAGGTATCAGATACTGATAGTGCAATTATTGATTTAGGAACTATAAATCAAAATAGGGTTGCTTATTATGTAAATGGAGCTAATAGTATTTTAAGAGATAAAGGAAATAATACTATTGGAAAAATTACTGGATATGGAGTTGGTGTATATCTTGAAGGAAATGCCTCAAAATCAACAGTAGGAATAGCAAAATTAGATGGAAGTACTCCAAAATTACATTATAAAAATTCAAATTTAGGAACTACTGGAAATGGGATAATTGGATTGTTCCTAAGTGGAGATACAGAAATTAAAAACTATACTAGTGGTATTGTAGTTGGAAATACAGTTGAAGAAAATTCTTTAAAGAAATATGCAATAGGTATCTATGCAGATAAACAAGGAACTTCAACAAATAAATATGAAATTAATACTGATATAACAACTGGTAAAGAGGGAGTAGGTATTTACTCTGATAATAATAGTGTTATAAATTATAAAGGAAAAATAGCAGTAGGAGATGGTTCAGTAGCAACAAAAAATGATGAAGTAACTGCTGGTATAGGAATTTATATCACTAAAAGTACTGGTGGAAATATTGGAGAGGTTAATTTAATTGGAAGCAAGACAGATACAAGTATTACATTAAAAGGAACAGGAGGAGTTGCAGTTATAGCCTCTGAGGGTACAAGGTTTAATGGTGGAAATGCTACAATTAATCTAGTAGGAACTAATATAAAAGGTGTAGGAGTTTATGCTAAGAGAGGTTCAGAAGTTAAAATAGATAACTGGACTTTTAATAATAATGGTAATTCAGCAGAGGAAGTTCGTTCAGAAGAAGGAGGGGCTCATATTACTGCTACTATTAAACAATTAAAACCTAAAATGGTTTTAAGCCATGTTATTAATGGAGAAACTTCTATTGCTGCTGGAAGTAAAGTTGTTTCTGTTGATGATGCTCCACATAGAGCAGCTGAAAATATAGGACTTATGGCAGAAGGAAGAAAAAATCCAACTGCACCAGCACCACTTACTGCTTGGACAAATGGAGATTTTGAAATAGAAAATCATGGAACTATTGATTTTTCAGTAGCTGAGAAATCAACAGCAATATTCTCAAATTCTGCTAGAGCTAAAAATGATGGAATAATTAAAGTTGGGAAAAATTCAACAGCTATTTATGGTTTCTATGATTCAACAATAAGAACTTATGAAGTTGTACCACCAGCAGTGGCTTTACCTAATAAGTTAGAAATTGAAACAACTTCAAATTCTAAAATAAGCTTAGGTGATAAATCAACAGGAATGTATTTAATAAATGCAGAAAAAGTAGAAAATAAAGGTGGACAAATAACTTCTGAAGTAGGAGCTACAAAGAATGTAGGTATCTATGCAATAAATGGAGCAGTTGATAAAGGAAATGCCAAAGATAATTCTATTTATAATAAGTCACAAAACTATAAAACTTTAAATATGGTAACTGCTACTAATATAACATTAGGAAATGGTTCAGTTGGATTATATAGTAAAGGGCAATCTAATAATGTTAGAAATAGTGTGACAAATACAGGAAATATAACAGTAGGAAGTAAAATAACAGTTAGTAAAACTGAAAATTATCCATCAGTTGCAATGTATGCTGAAAATACAAACTTAAATACTAATTCAAATGTAACAGTTGGTAATGATGGAATAGCTTTCTATGGAAAAAATTCAGATATTACTGCAAAAGGTAGTGTAAACTTCTCAAATAATGGAGTACTTGCATATTTAGAAAATTCTAAATTTGTTTCTCATTTAGGAAATTTAGGAGCAACAAAAAATACTATGATGTATTTAAAAAATAGTATAGCTCAATTAGATGGAGCAGGAACTAAGGTAGATGTAGAAGTTGCTGATAACTATACAGGAGCATATATTGAAGGAAATTCTCAATTAACAGGGATAAAGACAATAAAACTAGGAGAAAATTCTACTGGACTTTATTTAAAAGATACAATGCCTAATTTTATTTCAACATCAGAATTAATAATTGGAACAAAAGATAAGGCAAGAGGAATTCTAGGAGTAAATTCTAATTTTACAAATAATAGTAAAATTAATTTAAGTGGAGTAGAATCGATAGGAATTTATTCTAATGCAGGCAGTGATAAAACTGTTGTTAATAATGGAGAATTAACTTTATCTGGAAAACAAACATTAGGGGTATTTTTAAGAGGTACACAAAATTTTGAAAATAAAGCTAACATAAATATAGCTAATTCAGTTAATTCAAAATATCCAACTATTGGAATTTATACAGCTACAAGTACAGTTGGTGCTAGAATTTTAGAAGGAGCTAATATAAAGCATACTTCTGGAACAATAGAAGTTGGACAAAAATCAATAGGTATTTATTCAAAAACTAGCTCAAATGTTGAAGTAAGTGCTGGAAAAATCCATGTTAAAGATCAAGGTATAGGAATCTATAAACAAAATGGAAAAGTATCTATAAAAGGTATATTAGATATAGATAAACATACTGCAACAGTAAAAGACAGTGAACCTACTGGTGTGTATGCTGTAAATGGAGCTCAAGTTGATGATCAAGCTTCTAAAATTTCTATAGGTGCAAAATCTTATGGATTTATCTTAAATAATACTGATTCAACAAAGACTAATACTTATAGTAATAGAAACACAGGAACTGTAAGTTTAGGAAATGATAGTGTATTCCTATATTCTAATGGAAAAGCAAGCATTGTTAATAATAGAACAATAAATTCAAATGGCTCTGAACATTTAATTGTTTTCTATATTAAAAATGGTGGAGATTTCACAAACAATGGAACAATAAATTTCTCAACAGGAAAAGGAAATATAGGAGTTTATGCACCAGGAGGAAAAGCAACAAATAAAGGTAATATTTTTGTTGGAAAAACTGATGATATAGATCCAGCAACAGGAAAAGTATATTCAGATGTTTCAAAAATTGTTTATGGTATAGGAATGGCTGCTGATAATAGTGGACATATAGTAAATGATGGAACAATTAGAATATACAATAATAAATCTATTGGTATGTATGGTTCAGGAATTGGAACAGTTGTAGAAAATGGAGTTAATGGAAAAATTCTTTTAGATGGAAGTAAAGCAACTGCAACTGATAAAATTCAAAGTATGACAGGAGTTTATGTTGATGAAGGAGCTACATTCAGAAACTTTGGTACTATAACAACAACAGATTCTTATGCTGGAAGAAATGGAAAAGTAAATGAAAATGTTTCAGGACTTACTGGTGTTGCAGTAATGAATGGTTCAACTCTTATAAATGAATCAACAGGAAAAATCTTAATTGATGCTGATAATAGTTCAGGAGTTGTTATTAGAGGAAAAAGAGATGCTAATGGAAAATTGGTAAGAAATGCTGTAATTAAAAACTATGGAGAAATTAGAGTAAGAGGTAAAGGTACAACAGCTATTAGTTGGAAAGATGTTAGTCCAGCAGATATTGCTGAATTACAAAAACAAATTAATGATAAAATTACTTCAGATCCTAGCGGACGTGAATTAGGACAAGCTAGTGGTACTAATAAAGAATACCAAGGAGTTACAATCACTGTAAAAAATGGAAAACCTGTATTTACAAGAAATGGAAAATTAATTTCTGATAGTGAAGTTGAACAAATTAATAAGCTGATAGGAAGTGCACCAAACCTTGCAATGTCTGATGTTGGTTTCTATGTTGATACATTAGGAAGAACAAAACCTGTTACATTTGATGGAGCAAATCCACCTGTAAATAGCCAATTGATTATTGGTACTGAGTATTCAGAAAAAACTAATAAGAAAGAATGGTTTGTAAGTGGAAATGTAATTAAACCTTTCCTAGATCAAATCCAAGGAAGAAACTTTAAATTAACAACATTAGCTGGTTCATTAACTTGGATAGCTACACCAGTTTTAGATAACTATGGACAAATAACAGGTGTTGGAATGTCTAAATTAGCTTATACATCTTTTGTAAAAAGAGAAGATAATGTATACAACTTTACAGATGGTTTAGAACAAAGATATAATGTGAATGCTATTGATTCAGTAGAAAAGAGAATTTTTAATAAATTAAATGGTATTGGTAAAAATGAAGAAGTATTATTAACACAAGCATTTGATGAAATGATGGGACACCAATATGCAAATGTACAACAAAGAGTACAAGCAACTGGAAATATTTTAGATAAAGAATTCTCTCATTTAAGAGGTTCTTTGGCAAATGCTTCAAAAGATTCTAATAAAGTAAAAACTTTTGGAATGAAAGGTGAATATAAGACAGATACTGCTGGTGTATTAGATTATAAGAACAATGCATATGGAGTAGCTTATGTTCATGAAAATGAAGATATCAAACTTGGAAAAGGTACAGGTTGGTATACAGGTATAGTTCATAATACTTTCAAATTTAAAGATATTGGAAACTCAAAAGAAGAACAATTACAAGCTAAGGTTGGATTATTTAAATCAGTTCCATTTGATGAAAATAATAGCTTAAATTGGACAATATCAGGAGATATCTTTGTTGGACACAATAAACTAGAAAGAAAATTCTTAGTTGTTGATGAAATATTCCATGCAAAATCTAAATACTATACTTATGGAATAGGAGTTAAAAATGAAATAGGAAAAGAATTTAGATTAAGTAAGGCTTCTCAGTAAGACCTTATGCAGCATTAAAAGTTGAATATGGAAAAGTGTCTAAGATAAAAGAAAAATCTGGTGAAATGAAATTAGAAGTTAAAGAAAATGACTACTTATCTATAAGACCTGAAATAGGAACAGAACTAGCATATAAACATTACTTTGGAACTAAATCTTTAACTGCATCAGTTGGAGTAGCTTATGAAAATGAGCTAGGAAGAGTTGCAAATGGAAAGAATAAAGCAAGAGTAGCAGGAACAACTGCTGATTGGTTTAATATCCGTGGTGAAAAGGAAGATAGAAAAGGAAATGTAAAAGTTGACTTGAATGTTGGACTTGATAACCAAAGATTTGGAGTAACTGGAAATGTAGGTTATGATACAAAGGGAAGCAATGTTAGAGGTGGAGTAGGACTAAGAGTTATATTCTAA |
| Amino acid sequence of virulence factor Fap2 of Fusobacterium nucleatum | MEAPEIKPEPNKPIEAPKITLPTINKVIIEELNITPPAALNVPGTPNINITINAPNAPTPPSVSVNVSEPSAPNAPNINIPITPPEIKALNIETPPTVNVAPPTVAAINPVAFSVAPTIDSKQYKFGGANINNGLNGLPSTIDVQSNSNTNRNYVTINASGSSVPTSVLLPDKKTINVSVNNNRALVVDEARENFDFQMKGTINLYGNQNMGIDLQGTHIGGSAKGSATNPAISTIRNEGVITGHATNKYDNNKATKEQVAFGFSNADASSNATMTHMINNNEITLNAPSSAGIQLKPEDPFYWDPNWGTLSSDNKMIINGLSGSRNANNFGRVLMKADNRKDINLNGSGSFGMITVFNPGVIELNTISVNRTTYPTGSYNLRAQRNIGTKVLPGGEIGRSALSDSKYTSGVYNSGNININGDESVGVGILHEIQEVKIGGTINIGVESVSQTTGISDTKSTTGNDQTTLVNNAVGVFSGVPTLPVKNNEYDTMGNKNTTGNIIGTETSEVDGTINIGRHSKESIGLLVGDSGETLNNGTLNGTPNQARILKRSGSITYKSSANNKLNINGNANYGFVVKSDSNKSVFGSALDDLQQSVDKTNYGIGINKGNIDIIGINSIGFVLLKGGNSKNTGNITVNESEDFWYPIVPPTPNYDYRQNSIGFYGEQDNFTNEGTISVNTPNRSGNKAVLLKGNSNGITFNNTGDVSVKGRNNIAIYAEGKYTFNHEKNAAGTNKISVGSNSIGIYVKDNTGTVNIKAPIELADSSNGTTIGVYSDGNAHINFENGSKLTIGKKAIGLFSQSTTNFKNTFKFNNTAGNELNVSLDENSAFAFFNGSGSTDIAEVLNKNIKFTAMKKGATFAYVKGGSIVTLSHDFDTTNTAKVVVAPESGTSVLVASDGSTVQVDANKKLTTNTNVGLVATNGSSSNKSKAINKGTIISKVNGGVGLYANNNSEATNDIAGKITMENKGSAAILGENNSILTNKKDIELKEEESAGIYAKDSLATNSVTSGSPAVKAKISLEKTKSAGMYSILTNLADGDKKILNQANGADKASIEIGANATSSAGMFGKVESNATKVLTLENAGNIQVGATSSVGMYAQNETVDEDKLLVNNTGLINVTKESSVGINASKRATITNSGVTADGNGIVLSAKKTAGIIGNNNSVVTNTGDITLNSTTQPTDSSEGLVGISVDKTSTALNNGTITVSNDYSTGISGKGGRITNNNKIILNNINSVGISSTDGSVLNSADTNNLIEVKNSNSVGIFAKLIENVPFSPFQAILNKGTITLNGGNSKSSSVGIYSLIEDKAVQNKSTQQIANTGTINVSTKGSAGIYVKDNRTSGTDVISFIRNEKDINVAEESSAGIIGEYSQITNGNPQNTNGKIELSAKKTAGIIANKKSEVINYGKIETLSSVTINNKTDALVGISVNNSKVINKNKEDSNGMIGAITLNTAYSTGIYGKNESTLINEGKITANKTDSIGMAGENSSLTNKNEILINEEKSVGMFGTTVGNKNTVLINEQDDIKSLTGKIITKKKESVGMFVSTSQASAINRGTILIEEEKSAAMYGDEANIENSGTSTNLIASITTKKDESAGIYAKNSNATNKKKMTVEGKKSAAILVEMTKDKNISGINDIADNSSDILNGHIEVKNQESAAMYGKIDSKVSVATSTLTLINKKAIDIDAKSSVGMMLINDSNSVNKTNVKAENSGVITLKNATNNTGNIGILAKKNSTGINKDNGIINVNSKKSIGMLAKEGSNVENNSNLPPNPLVVGKEYGINLNEEESIGMLAEGQNSASQVSTAINNAKISVTAAGKKSIGMLAQNEGDVKNNKEIEVFAEKGVGIFVSDTGTGENTNPNGKITLMNKEAVGIFAKNNGDIYTAKNSGKVILGSADGTTTHESLIGMFAQAEAGKKSSIKNIDTIDVNTKKSVGMYAKNDAANVDDVDLYNAGTINVNNKGSAGIYAPKANISKVGNIKLKNTTDTDGASAVYISQGGKVSDTDSAIIDLGTINQNRVAYYVNGANSILRDKGNNTIGKITGYGVGVYLEGNASKSTVGIAKLDGSTPKLHYKNSNLGTTGNGIIGLFLSGDTEIKNYTSGIVVGNTVEENSLKKYAIGIYADKQGTSTNKYEINTDITTGKEGVGIYSDNNSVINYKGKIAVGDGSVATKNDEVTAGIGIYITKSTGGNIGEVNLIGSKTDTSITLKGTGGVAVIASEGTRFNGGNATINLVGTNIKGVGVYAKRGSEVKIDNWTFNNNGNSAEEVRSEEGGAHITATIKQLKPKMVLSHVINGETSIAAGSKVVSVDDAPHRAAENIGLMAEGRKNPTAPAPLTAWTNGDFEIENHGTIDFSVAEKSTAIFSNSARAKNDGIIKVGKNSTAIYGFYDSTIRTYEVVPPAVALPNKLEIETTSNSKISLGDKSTGMYLINAEKVENKGGQITSEVGATKNVGIYAINGAVDKGNAKDNSIYNKSQNYKTLNMVTATNITLGNGSVGLYSKGQSNNVRNSVTNTGNITVGSKITVSKTENYPSVAMYAENTNLNTNSNVTVGNDGIAFYGKNSDITAKGSVNFSNNGVLAYLENSKFVSHLGNLGATKNTMMYLKNSIAQLDGAGTKVDVEVADNYTGAYIEGNSQLTGIKTIKLGENSTGLYLKDTMPNFISTSELIIGTKDKARGILGVNSNFTNNSKINLSGVESIGIYSNAGSDKTVVNNGELTLSGKQTLGVFLRGTQNFENKANINIANSVNSKYPTIGIYTATSTVGARILEGANIKHTSGTIEVGQKSIGIYSKTSSNVEVSAGKIHVKDQGIGIYKQNGKVSIKGILDIDKHTATVKDSEPTGVYAVNGAQVDDQASKISIGAKSYGFILNNTDSTKTNTYSNRNTGTVSLGNDSVFLYSNGKASIVNNRTINSNGSEHLIVFYIKNGGDFTNNGTINFSTGKGNIGVYAPGGKATNKGNIFVGKTDDIDPATGKVYSDVSKIVYGIGMAADNSGHIVNDGTIRIYNNKSIGMYGSGIGTVVENGVNGKILLDGSKATATDKIQSMTGVYVDEGATFRNFGTITTTDSYAGRNGKVNENVSGLTGVAVMNGSTLINESTGKILIDADNSSGVVIRGKRDANGKLVRNAVIKNYGEIRVRGKGTTAISWKDVSPADIAELQKQINDKITSDPSGRELGQASGTNKEYQGVTITVKNGKPVFTRNGKLISDSEVEQINKLIGSAPNLAMSDVGFYVDTLGRTKPVTFDGANPPVNSQLIIGTEYSEKTNKKEWFVSGNVIKPFLDQIQGRNFKLTTLAGSLTWIATPVLDNYGQITGVGMSKLAYTSFVKREDNVYNFTDGLEQRYNVNAIDSVEKRIFNKLNGIGKNEEVLLTQAFDEMMGHQYANVQQRVQATGNILDKEFSHLRGSLANASKDSNKVKTFGMKGEYKTDTAGVLDYKNNAYGVAYVHENEDIKLGKGTGWYTGIVHNTFKFKDIGNSKEEQLQAKVGLFKSVPFDENNSLNWTISGDIFVGHNKLERKFLVVDEIFHAKSKYYTYGIGVKNEIGKEFRLSEGFSVRPYAALKVEYGKVSKIKEKSGEMKLEVKENDYLSIRPEIGTELAYKHYFGTKSLTASVGVAYENELGRVANGKNKARVAGTTADW  FNIRGEKEDRKGNVKVDLNVGLDNQRFGVTGNVGYDTKGSNVRGGVGLRVIF |
| Similar nucleotide sequence of virulence factor Fap2 of Fusobacterium periodonticum | CTAATATAAAACATACATCTGGAACAATAGAAGTTGGTGAAAAATCAATAGGTATCTATTCAAAAACTCCTTCAAGTGTTGAAATGAATGGTGGAAAGATACATGTTAAAGATCAAGGTATAGGAATCTATAAAGAAGATGGTACAGTTGTTGTAAAAGGTGAATTAGATATAGATAAACATGTGGCAACAGCAAAAGATACAGAACCTACTGGAGTTTATGCAGTAAATGGAGCAACAGTTGTTGATCAAGCTTCAAAAATTACAGTTGGAGAAAAATCTTATGGATTCATATTAAATAATACTGATCCAAATAAAACAAATGTTTATACTAATACAAATGCAGGACCTGTAAGTTTAGGAAATGATAGTGTATTCCTATATTCTAAAGGAAAAGCTAACATCACTAATAATAGAAATATTAATTCTAATAATTCAGATCACTTAATAGGATTCTATATTAAAGATGGTGGAGATTTCGTAAATAATGGAATAATAGACTTCTCAACAGGAAAAGGAAATATAGGAGTTTATGCACCTAATGGAAAAGCAACAAATAGAGGAAGCATTGTTGTAGGACCTACTGATGATATAGATCCAGCAACAGGAAAAGTGTATTCAGATGTTTCAAAAATTGTTTATGGTATAGGTATGGCTGCTGATAATGGTGGACATATAGTAAACGAAGGAGATATTAGAATATCTACTAATAAATCTATTGGTATGTATGGTGCAGGAATTGGAACAATTGTAGAAAACACAGGAAGAATCTTATTAGATGGAAGCCAAGCTACAGCAACTAATAAAATTCAAAGTTTAACAGGAGTCTATGTTGATGAAGGAGCAACTTTCAAAAATAGTGGGCTTATCACAACAACAGATTCTTATGCTGGAAGAAATGGAAAAATAAATGAGAATGTAACAGGACTTACTGGAGTTGCAGTAATGAATGGTTCAACTCTTATAAATGAAGCAACAGGAAAAATCTTAATAGATGCTGACAACAGTACAGGAGTTGTTATCAGAGGAAAAAGAGATGCTGCAGGAAACTTAATAAGACCAGCAGTAATTAAAAACTATGGAGAAATTAGAGTAAGAGGTAAAGGTGGTTCAGCAATCAGTTGGAAAGATGTTAGTGCTGCAGATATTGCTGAGTTAGAAAGACAAATTAACTCTAAGATTACAACAGACCCTTCTGGAAATGAAATAACTCAAGCTAGTGGAACAAGTAAAGATTACCAAGGAATCACTATCACTGTAAAAAATGGTCAAGCTACATTCTTAAGAAATGGAGTACCAGTATCAGATAGTGAAGTAGAAAAAATTAATAAGTTAATTGGTAATGAACCAAATCTAGCAATGTCTGATATAGGATTCTATATAGATACATTGGGAAGAACAAGACCTGTTACTTTTGATGGAGCAGCCCCACCAGTAAATAGCCAATTAATAATTGGTACTGAATTCTCAGAAATGACTAATAAAAAAGAATGGATAGTAAGTGGAGATGTTATTAAACCTTTCTTAGATCAAATTCAAGGAAGAAACTTTAAAATAACAACTATGGCTGGTTCATTAACTTGGATGGCTACACCAATCTTAGATAACTACGGACAAATTGTAGGAATGGCAATGTCTAAATTAGCTTATACATCTTTTGTAAGACCAGAAGATAATGCATATAATTTTGCTGATGGATTAGAACAAAGATATGATATGAATGCTCTTGATTCAGCTGAAAAGAGACTATTCAATAAATTAAATGGAATCGGAAAAAATGAGGATGTATTATTAACTCAAGCTTATGATGAAATGATGGGACACCAATATGCTAATGTTCAACAAAGAATACAAGCAACAGGAAGAATATTAGATAAAGAATTCTCTTATTTAAGAAATTCTTGGTCTAACCCTTCAAAAGATTCTAATAAGGTAAAAACTTTTGGAATGAAAGGTGAATATAAAACAGATACTGCTGGTATAATAGACTATAAGAACAATGCTTATGGAGTAGCTTATGTACATGAAGATGAAACAGTTAAGTTAGGAGAATCTACAGGTTGGTACACAGGTATAGTTCATAATACTTTCAAATTTGAAGATATTGGAAAATCAAAAGAAGAACAATTACAAGCTAAGGTTGGATTATTTAGATCAGTACCATTTGATGATAACAATAGCTTAAATTGGACAATATCAGGAGATATTTTTGTAGGACATAACAAACTAAACAGAAGATTCTTAGTTGTTGATGAAATATTCCAAGCAAAATCTAAATACTATACTTATGGAATAGGAGTCAGAAATGAAGTAGGAAAAGAATTCAGATTGAGCGAAGGACTTTCAGTAAGACCATATGGAGCAGTGAGAGTTGAATATGGAAGAATGTCTAAGATAAAAGAAAAATCTGGTGAAATGAAATTAGAAGTTAAATCAAATGACTACTTATCTATAAGACCAGAAGTAGGAACAGAATTAGCATATAAACTTCACTTAGGAAATAAAACATTGAGAGCAGCCCTAGCAGTAGCTTATGAAAATGAGTTAGGAAGAGTTGCAAATGGAAAGAATAAAGCAAGAGTTGCTGGAACATCTGCCGATTGGTTCAATATCAGAGGTGAAAAAGAAGACAGAAGAGGAAATGTTAAAACAGATCTTAATGTTGGTGTAGATAACCAAAGAATTGGAGTAACTGGAAACGTAGGTTACGATACAAAAGGTAGAAATGTTAGAGGTGGCTTAGGACTAAGAGTTATATTCTAATTTAAAAATTTAAATTATAAGAGCTGATTGAAGTGAAAACTTCAATCAGT |
| Similar amino acid sequence of virulence factor Fap2 of Fusobacterium periodonticum | NIKHTTGTIEVGEKSIGIYSKTPSSVEMNGGKIHVKDQGIGIYKEDGTVVVKGELDIDKHVATAKDTEPTGVYAVNGATVVDQASKITVGEKSYGFILNNTDPNKTNVYTNTNAGPVSLGNDSVFLYSKGKANITNNRNINSNNSDHLIGFYVKDGGEFVNNGIIDFSTGKGNIGVYAPNGKATNRGSIVVGPTDDIDPATGKVYSDANKIVYGIGMAADNGGHIINEGDIRISTNKSIGMYGAGAGTIVENTGRILLDGSQATATNKIQSLTGVYVDEGATFKNSGLITTTDSYAGRNGKINENVSGLTGVAVMNGSTLINEATGKILIDADNSTGVVIRGKRDAAGNLVRNAVIKNYGEIRVRGKGGSAISWKDVSAAEIAELERQINSLISTDPSGNEITQASGTSKDYQGITITVNGQATFLRNGVPVSDSEVEKINKLIGNEPNLAMSDIGFYIDTLGRTRPVTFDGAAPPVNSQLIIGTEFSEMTNKKEWIVSGDVIKPFLDQIQGRNFKITTMAGSLTWMATPILDNYGQIVGMAMSKLAYTSFVRPEDNAYNFADGLEQRYDMNALDSAEKRLFNKLNGIGKNEDVLLTQAYDEMMGHQYANVQQRIQATGRILDKEFSYLRNSWSNPSKDSNKVKTFGMKGEYKTDTAGIIDYKNNAYGVAYVHEDETVKLGESTGWYTGIVHNTFKFEDIGKSKEEQLQAKVGLFRSVPFDDNNSLNWTISGDIFVGHNKLNRRFLVVDEIFQAKSKYYTYGIGVKNEVGKEFRLSEGLSVRPYGAVRVEYGRMSKIKEKSGEMKLEVKSNDYLSIKPEVGTELAYKLHLGNKTLRAALAVAYENELGRVANGKNKARVAGTSADWFNIRGEKEDRRGNVKTDLNVGVDNQRIGVTGNIG |
