# Supplementary figures and images for: Fusobacterium periodonticum BCT protein targeting glucose metabolism to promote the epithelial-mesenchymal transition of esophageal cancer cells by lactic acid
Source: J Transl Med. 2024 Apr 30;22:401. doi: 10.1186/s12967-024-05157-z (PMC11061911; doi:10.1186/s12967-024-05157-z)

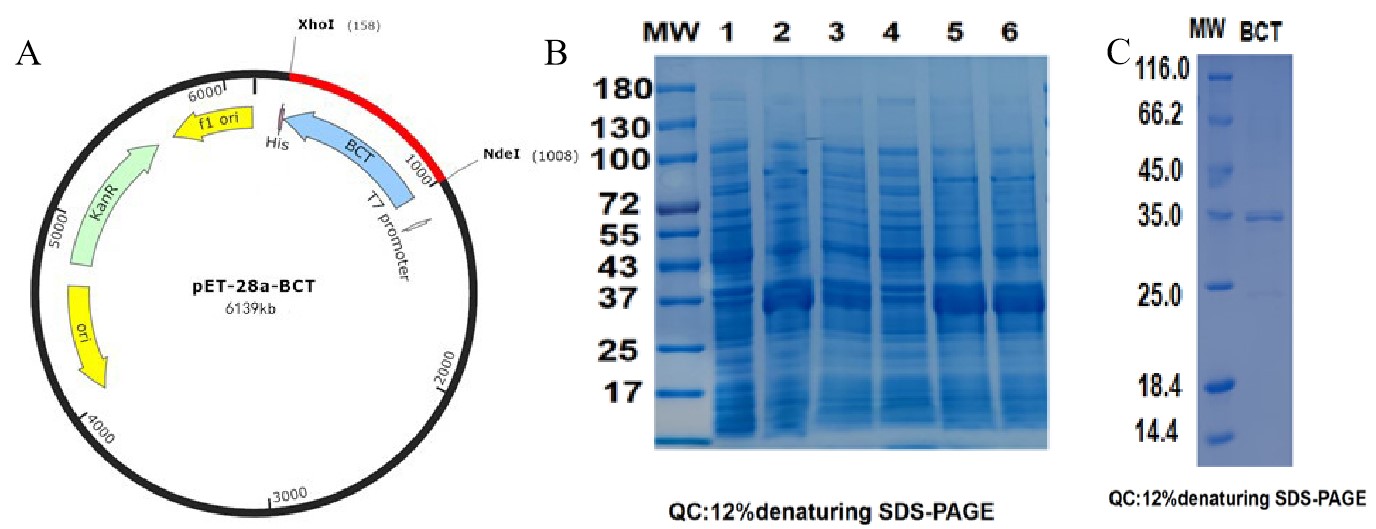

Supplement: Supplementary file 2 — Supplementary Material 2 [file 12967_2024_5157_MOESM2_ESM.jpg]
